# Hepatocyte-Derived IL-25 Promotes Macrophage Extracellular Trap Formation and Drives Liver Fibrosis Progression

**DOI:** 10.1007/s10753-026-02506-6

**Published:** 2026-04-01

**Authors:** Cheng-Jiang Cao, Ming Yang, Chang-Lin Du, Xue Fang, He-Hang Song, Wen-Jing Wang, Miao-Miao Wang, Wen-Mei Zhang, Zhen-Long Liu, Cheng Huang, Jun Li

**Affiliations:** 1 Inflammation and Immune Mediated Diseases Laboratory of Anhui Province, Anhui Institute of Innovative Drugs, School of Pharmaceutical Sciences, Anhui Medical University, Hefei, 230032 China; 2 School of Pharmaceutical Sciences, Anhui Medical University, Hefei, China

**Keywords:** Liver fibrosis, Interleukin-25, Macrophage extracellular traps, Citrullinated histone H3, ROS, NOX, Mitochondrion, Lysosome

## Abstract

Liver Fibrosis (LFib) represents a significant global health burden as a chronic progressive disease. Emerging evidence has established a critical link between macrophage extracellular traps (METs) and LFib; however, the precise triggers of METs formation and their mechanistic contributions to fibrosis remain poorly understood. Substantial evidence indicates that interleukin-25 (IL-25) potently regulates macrophage metabolism and LFib progression. A carbon tetrachloride (CCl₄)-induced mouse model of liver fibrosis spanning distinct stages (2 to 8 weeks) was established. In vitro studies utilized co-culture systems and conditioned medium treatment to assess METs-mediated activation of hepatic stellate cells (HSCs). To elucidate the specific role of IL-25, hepatocyte-specific IL-25 conditional knockout (IL-25CKO) mice were generated using CRISPR/Cas9 technology and subjected to the LFib model. Mechanistic investigations involved stimulating RAW 264.7 macrophages with rmIl-25 and specific inhibitors. Techniques such as scanning/transmission electron microscopy, immunofluorescence, Western blot and ELISA were employed to analyze MET formation, ROS production, lysosomal activation, mitophagy, and related signaling pathways. Fibrotic livers exhibited strong early co-localization of IL-25 with hepatocytes, which preceded METs formation. This demonstrates that early production of IL-25 by hepatocytes acts as a triggering factor for METs formation. In vitro co-culture systems revealed METs-mediated activation of HSCs, while mechanistic studies showed that IL-25 promotes IL-17RB receptor activation, triggering downstream reactive oxygen species (ROS) bursts, lysosomal activation, and METs formation. This work identifies a pathogenic hepatocyte–macrophage–HSC axis and supports IL-25 early blockade as a promising clinical strategy for LFib.

**Supplementary Information:**

The online version contains supplementary material available at 10.1007/s10753-026-02506-6.

## Highlights

Hepatocyte-derived IL-25 drives macrophage extracellular trap formation and liver fibrosis.

IL-25 induces METosis through IL-17RB, triggering oxidative stress and organelle dysfunction.

METs activate HSCs and promote fibrogenesis.

Hepatocyte IL-25 knockout or anti-Ly6g-DNase I therapy inhibits MET-driven fibrosis in vivo.

Targeting the IL-25–MET axis represents a promising therapeutic strategy for liver fibrosis.

**Supplementary Information:**

The online version contains supplementary material available at 10.1007/s10753-026-02506-6.

## Introduction

LFib, a maladaptive repair process triggered by chronic hepatic injury, represents a critical pathological pathway in various chronic liver diseases, including viral hepatitis, alcohol-related liver disease, and non-alcoholic steatohepatitis (NASH) [1]. Characterized by excessive deposition of extracellular matrix (ECM) components, this progressive scarring disrupts hepatic architecture and function, ultimately leading to cirrhosis, portal hypertension, and hepatocellular carcinoma if left unmanaged [2]. Central to fibrogenesis is the activation of HSCs, which transdifferentiate into myofibroblasts under inflammatory and oxidative stress, driving collagen synthesis and ECM remodeling [3]. This process is further modulated by crosstalk among hepatocytes, immune cells (e.g., macrophages), and ROS [4].

Macrophages, as versatile regulators of innate immunity, play dual and context-dependent roles in the pathogenesis and resolution of liver fibrosis [5]. While resident Kupffer cells are traditionally recognized as the primary macrophage subset in the liver, recent evidence underscores the critical contribution of bone marrow-derived macrophages (BMDMs) to the pathogenesis of liver fibrosis [6], particularly under chronic injury conditions. This study aims to investigate the inhibitory effects on fibrosis progression by targeting inflammatory activation in macrophages.

Recent advancements in immunology have unveiled a novel antimicrobial mechanism employed by macrophages: the release of METs [7]. Analogous to neutrophil extracellular traps (NETs), METs are web-like structures composed of decondensed chromatin scaffolds decorated with granular and cytosolic proteins, including histones and antimicrobial peptides, which immobilize and neutralize invading pathogens [8]. While NETs have been extensively studied, METs remain relatively undercharacterized, with emerging evidence highlighting their dual role in host defense and immunopathology [7, 9]. METs are implicated in trapping bacteria, fungi, and parasites, thereby limiting microbial dissemination [10]. However, excessive or dysregulated METs formation has been associated with chronic inflammatory diseases, autoimmune disorders, and tissue damage due to the cytotoxic and pro-inflammatory properties of extracellular DNA and associated proteins [7]. The molecular triggers and signaling pathways governing METosis (METs formation) are distinct from those of NETosis, involving cytokine stimuli and ROS-dependent or independent mechanisms [11]. Coiled-coil domain containing protein 25 (CCDC25) is a plasma membrane-localized transmembrane protein that functions as a specific sensor for NET-DNA, promoting the distant metastasis of tumor cells. Studies have confirmed its role as an extracellular trap (ET)-DNA receptor on cancer cells, where it detects external DNA and subsequently activates the ILK/β-parvin pathway to drive cancer cell proliferation. In non-oncological contexts, research has also demonstrated that CCDC25 mediates ILK signaling, contributing to eosinophil extracellular trap (EET)-associated asthmatic responses. Given its established roles, we speculate that CCDC25 may similarly participate in the inflammatory processes induced by METs, a hypothesis that warrants further investigation [12].

ROS, long recognized as critical mediators of antimicrobial defense and cellular signaling, occupy a central role in macrophage immunobiology. These redox-active molecules, generated primarily via NADPH oxidase (NOX) activity and mitochondrial respiration [13], not only directly neutralize pathogens but also orchestrate immune responses by modulating inflammatory pathways [14]. In macrophages, ROS production is intricately linked to lysosomal function—a dynamic organelle system responsible for pathogen degradation, antigen processing, and cellular homeostasis [15]. Lysosomes contribute to ROS generation through enzyme-catalyzed redox reactions, while simultaneously serving as targets of oxidative stress, which can destabilize lysosomal membranes, trigger enzyme leakage, and amplify inflammatory cascades. Emerging evidence implicates ROS as a pivotal regulator of METs formation [10]. Under excessive ROS stimulation, persistent damage to lysosomes within macrophages leads to lysosomal rupture and subsequent calcium ion leakage. As calcium ions activate peptidylarginine deiminase 4 (PAD4) [16], this leakage triggers PAD4 activation, thereby promoting chromatin decondensation and ultimately culminating in METs formation [17, 18]. Despite these advances, the tripartite relationship between ROS, lysosomal dynamics, and METs remains poorly defined.

Tissue stress or dysfunction induces inflammation, which helps tissues adapt to noxious conditions and restore functionality [19]. In the context of LFib, hepatocytes, which constitute the major parenchymal component of liver tissue, secrete intercellular regulatory factors in response to environmental stress. Notably, interleukins emerge as pivotal mediators among these factors, actively participating in diverse inflammatory and immunoregulatory interactions between cells [20]. IL-25, a member of the IL-17 cytokine family, is a key mediator of type 2 immune responses, primarily implicated in host defense against helminth infections, allergic inflammation, and tissue repair processes [21, 22]. Produced predominantly by epithelial cells and a subset of immune cells, IL-25 signals through a heterodimeric receptor complex (IL-17RA/IL-17RB) to activate downstream pathways, including NF-κB and STAT6 [23], thereby orchestrating the recruitment and activation of macrophages. Recent advances have highlighted its dual role in both pro-inflammatory and regulatory immune modulation, though its mechanistic interplay with macrophage-mediated effector functions remains incompletely elucidated [24]. Emerging evidence indicates that IL-25 activates downstream JAK/STAT and MAPK pathways via its cellular receptor [25–27], driving autophagy induction and ROS bursts—a prerequisite for MET formation. Mechanistic parallels with NETs suggest that IL-25-mediated signaling may regulate PAD4 activity, a key enzyme implicated in METs generation due to shared structural and mechanistic features with NETs. This study demonstrates that hepatocyte-derived IL-25, elevated under inflammatory conditions, binds macrophage IL-25 receptors to trigger downstream pathways. This activation induces ROS bursts, promotes METs release, and subsequently activates HSCs, ultimately accelerating LFib.

## Materials and Methods

### Use of Reagent

10 μM NAC (N-Acetyl-L-cysteine) (Cat. No. 106425, Sigma-Aldrich, USA), 10 μM CQ (Chloroquine) (Cat. No. J64459.22, Thermo Scientific Chemicals, USA), 50 ng/ml IL-17RB blocker (3,6-Dithia-1,8-octanediol) (CAS: 5244–34-8, Santa Cruz Biotechnology, USA), 25 μM VAS2870 (Cat. No. HY-12804; MedChemExpress, NJ, USA), 1U/ml DNase I (Cat. No. EN0521; Thermo Scientific, USA).

### Mouse Models of CCl4-induced Liver Fibrosis

Male C57BL/6 J mice (aged 6–8 weeks, weighing 18–22 g) were acquired from the Experimental Animal Center of Anhui Medical University (Anhui, China). All animal experimental designs were reviewed and approved by the Institutional Animal Experimental Ethics Committee (LLSC20251049). Mice were acclimatized for one week before experiments. They were randomly allocated into 8 experimental groups (*n* = 10 per group): Vehicle Control (VC: non-modeled mice receiving olive oil and saline injections), 2-, 4-, 6-, and 8-Week Negative Control (2W, 4 W, 6 W, 8 W NC: modeled for respective durations with saline intervention), IL-25 Only Treatment (4W IL-25 only: modeled for 4 weeks with recombinant mouse IL-25 administration), 4-Week anti-Ly6G Treatment (4W anti-Ly6G: modeled for 4 weeks with CCl₄, and anti-Ly6G administration), and 4-Week anti-Ly6G-DNase I Treatment (4W anti-Ly6G DNase I: modeled for 4 weeks with CCl₄, anti-Ly6G and DNase I administration). Fibrosis was induced in modeled groups (2W NC, 4 W NC, 6 W NC, 8 W NC, 4 W anti-Ly6G and 4 W anti-Ly6G DNase I) by intraperitoneal (i.p.) injection of 10% (v/v) carbon tetrachloride (CCl₄) in olive oil, administered twice weekly. The VC group received equivalent volumes of olive oil and saline via i.p. injection. All control groups received saline i.p. injections. Recombinant mouse IL-25 (rmIl-25; dissolved in saline) was administered via tail vein (i.v.) injection to the 4 W IL-25 group at 0.5 µg/20 g, twice a week continuing throughout the 4-week modeling period. DNase I (dissolved in saline) (Thermo Scientific) was administered i.p. to the 4 W anti-Ly6G DNase I group at 5 mg/kg daily. Saline injections were administered to the corresponding negative control group (4W NC). Neutrophils were identified using an anti-mouse Ly-6G antibody (Clone 1A8, Cat. NO. 127631, BioLegend) at 10 µg/g, twice a week. After modeling, mice were euthanized via anesthetic overdose 48 h after the final CCl₄ injection. Blood and liver tissue samples were collected for analysis.

### IL-25CKO Mice Strategy and Models

The generation of liver-specific IL-25 knockout mice utilizes a Cas9-mediated conditional knockout (CKO) approach within the C57BL/6JGpt genetic background. The strategy involves employing CRISPR/Cas9 along with donor vectors to introduce LoxP sites flanking Exon1–Exon2 of the Il25 gene, which targets its sole transcript Il25-201 (ENSMUST00000037863.5). This creates a conditional knockout allele. Following screening of positive F0 mice, they are bred with wild-type C57BL/6JGpt mice to establish a stable Il25-floxed line. These floxed mice are then crossed with strains expressing Cre recombinase under a liver-specific promoter. Cre-mediated recombination removes the LoxP-flanked exons, producing a knockout allele specifically in liver tissues/cells. Several considerations are important during this process. The Il25 gene is located on chromosome 14; therefore, when breeding with other strains to generate double-gene knockout mice, care should be taken to avoid loci on the same chromosome. Additionally, insertion of the LoxP sites may potentially affect the 5′-end regulatory regions of neighboring genes Efs and Cmtm5. Relevant phenotypic references include studies showing that male homozygous Il25 null mice exhibit significantly elevated serum triglyceride levels. This experimental design was developed by GemPharmatech Co., Ltd. (Jiangsu Jicui Yaokang Biotechnology Co., Ltd.), with a design date of August 14, 2020. Contact information: 400–9660890.

For fibrosis induction, mice received i.p. injections of 10% CCl₄ in olive oil twice weekly for 4 weeks. For the IL-25 rescue experiment, rmIL-25 was administered via i.v. injection at a dose of 0.5 µg per 20 g of body weight, twice per week.

### Serum Transaminases Assay

Blood was collected from each group to prepare serum. Alanine aminotransferase (ALT) (C009-2–1, Jiancheng Bioengineering Institute, China) and aspartate aminotransferase (AST) (C010-2–1, Jiancheng Bioengineering Institute, China) levels were measured using commercial kits according to the manufacturer’s instructions.

### Cell Lines and Culture

The murine immortalized macrophage cell line RAW 264.7 (Cat. No. CL-0190, Wuhan, China) and AML-12 (alpha mouse liver 12) cell line was purchased from ProCell (Cat. No. CL-0602, Wuhan, China). The murine immortalized hepatic stellate cell line mHSC was obtained from BNCC (Cat. No. BNCC337647, BeNa Culture Collection). RAW 264.7 and mHSC were cultured in Dulbecco's Modified Eagle Medium (DMEM; Gibco) and AML-12 was cultured in Dulbecco's Modified Eagle Medium F-12 (DMEM-F12; Gibco) supplemented with 10% fetal bovine serum (FBS; Gibco, USA) at 37 °C in a humidified 5% CO₂ atmosphere.

### Isolation and Differentiation of Mouse Bone Marrow-Derived Macrophages (BMDMs)

Mice were euthanized by cervical dislocation or CO₂ asphyxiation. The femurs and tibiae were aseptically dissected, and all attached muscle and connective tissue were removed. The ends of each bone were cut with sterile scissors. The bone marrow was flushed from the medullary cavity using a syringe filled with cold sterile PBS or growth medium (without M-CSF) into a sterile petri dish. The marrow suspension was gently pipetted up and down to dissociate cell clumps, passed through a 70-μm cell strainer to remove bone fragments and debris, and collected into a centrifuge tube. The cell suspension was centrifuged at 300 × g for 5 min at 4 °C. The pellet was resuspended in 2–3 mL of RBC lysis buffer, incubated on ice for 2–3 min, and then neutralized with excess complete medium. Cells were centrifuged again as above. The cell pellet was resuspended in complete growth medium containing 20 ng/mL M-CSF. Cells were counted and seeded at an appropriate density (5 × 10^5^ cells/mL) in non-treated culture dishes or plates. Cells were cultured at 37 °C in a humidified incubator with 5% CO₂. On day 3 of culture, an additional 50–100% volume of fresh complete medium with M-CSF was gently added without disturbing the adherent cells. By day 6–7, a confluent layer of adherent, mature macrophages (BMDMs) had formed. The medium was aspirated, and cells were washed once with warm PBS. For detachment, cells were incubated with enzyme-free cell dissociation buffer or cold PBS containing 2 mM EDTA on ice for 10–15 min, followed by gentle scraping. The harvested BMDMs were ready for subsequent experiments.

### Cell Stimulation and Treatment

RAW 264.7 and BMDMs were stimulated with 100 nM phorbol 12-myristate 13-acetate (PMA; Cat. No. G2272-100UL, Servicebio) for 8 h and IL-25 (Cat. No. HY-P73198; MedChemExpress; NJ, USA) 50 ng/ml for 24 h to induce METs formation. DNase I was administered at a concentration of 1 U/mL 1 h prior to IL-25 stimulation. For IL-17RB blockade, cells were pre-incubated with IL-17RBb 50 ng/ml for 1 h prior to stimulation with IL-25. The inhibitor was present throughout the subsequent stimulation period. RAW264.7 were pretreated with VAS2870 25 μM for 30 min prior to stimulation, NAC 10 μM for 1 h before stimulation, CQ 10 μM for 2 h before stimulation. AML-12 was stimulated with 0.2% CCl₄ in culture medium for 12, 24, 48 h.

### Cell Migration Experiment

Scratch assay: mHSCs were first seeded in 6-well plates. When the cell density reached 100%, a scratch was made along the diameter of the well using a pipette tip. Suspended cell debris was washed away with PBS, and the cells were photographed under a microscope while recording the imaging positions. The medium was then replaced with serum-free medium and serum-free medium containing 5 μg/ml METs, respectively. After 24 h, images were taken again for documentation.

Transwell migration assay: mHSCs were first seeded into the upper chamber of an 8-μm pore size Transwell insert. After the cells adhered, the medium in the upper chamber was replaced with serum-free medium and serum-free medium containing 5 μg/ml METs, respectively. The lower chamber was filled with complete medium containing 5% serum. After 24 h of stimulation, the medium was discarded. The non-migrated cells on the upper surface of the membrane were gently removed using a cotton swab. The upper chamber was rinsed three times with PBS. Subsequently, the upper chamber was immersed in a lower chamber containing paraformaldehyde for 30 min for fixation, followed by three PBS washes. The upper chamber was then immersed in a lower chamber containing 0.1% crystal violet staining solution for 20 min. After staining, it was rinsed with PBS until no excess crystal violet remained. The migrated cells on the lower surface of the membrane were observed under a microscope.

### Histopathology and Immunohistochemistry

Liver tissue sections were fixed in 4% paraformaldehyde for 24–48 h, dehydrated through a graded ethanol series, cleared in xylene, embedded in paraffin, and sectioned at 5 µm thickness. Sections were stained with hematoxylin and eosin (H&E), Sirius Red (SR), and Masson’s trichrome (Servicebio, Wuhan, China) using standard methods. For immunohistochemistry, deparaffinized sections underwent antigen retrieval, blocking with 3% BSA, and incubation with primary antibody against α-smooth muscle actin (α-SMA; Servicebio) diluted 1:200. After washing, sections were incubated with HRP-conjugated secondary antibody, developed with DAB, counterstained with hematoxylin, and mounted. Slides were scanned using a Pannoramic MIDI scanner (3DHISTECH Ltd., Hungary) and analyzed with CaseViewer software. Histopathological evaluation was performed in a blinded manner. For each mouse liver tissue, one representative section was analyzed, six non-overlapping fields of view per section were randomly captured under a 20 × magnification. The positive stained area for α-SMA, Sirius Red, and Masson’s trichrome was quantified using Image J software. A consistent color threshold was applied to all images to identify positive signals, and the results were expressed as the percentage of positive area relative to the total analyzed tissue area. Quantifications were based on *n* = 6 biologically independent mice per group, yielding a total of 36 analyzed fields per stain per group. Data are presented as mean ± SEM.

### Immunofluorescence Staining

Cells were washed with PBS, fixed, permeabilized, and blocked with 5% BSA. mHSCs were incubated with anti-α-SMA (Cat. No. A7248, ABclonal) and anti-collagen I alpha (COL1A1) antibodies (1:100) (Cat. No. A24112, ABclonal), while RAW 264.7 cells were incubated with anti-CitH3 antibody (Cat. No. ab219407, Abcam). After washing, cells were incubated with fluorescently labeled secondary antibodies (Cat. No. GB25303, Servicebio), and nuclei were counterstained with Hoechst 33,258 (Cat. No. BL804A biosharp). For tissue staining, fresh liver samples were embedded, sectioned, fixed, blocked, and incubated with primary antibody combinations: anti-F4/80 (Cat. No. GB11027-100, Servicebio) and anti-CitH3 (Cat. No. ab219407, Abcam), or anti-HNF4α (Cat. No. GB115549-100, Servicebio) and anti-IL-25 (Cat. No. NB100-56541, Novus Biologicals). Secondary antibodies and DAPI (Cat. No. G1012, Servicebio) were applied before imaging with a fluorescence microscope. For immunofluorescence double-staining (e.g., F4/80 and CitH3), co-localization was assessed using the Image J Manders’ overlap coefficients were calculated to quantify the fraction of one signal overlapping with the other across at least 36 fields per group from 6 independent animals.

### CCK-8 Analysis

mHSC proliferation was assessed using the CCK-8 (Cat. No. 40203ES60, Yeasen Biotechnology, Shanghai, China) assay. Cells were seeded in 96-well plates, allowed to adhere, and cultured in MET-containing supernatant for 24 h. CCK-8 solution was added, and absorbance was measured at 450 nm.

### Western Blotting

Proteins were extracted using RIPA buffer with inhibitors. Concentration was determined via BCA assay. Proteins were separated by SDS-PAGE, transferred to PVDF membranes, blocked, and incubated with primary antibodies: α-SMA (Cat. No. A17910, ABclonal), COL1A1 (Cat. No. ab260043, Abcam), CitH3 (Cat. No. ab219407, Abcam), phospho-p47phox (Cat. No. AF3917, Affinity Biosciences), β-actin (Cat. No. GB15001, Servicebio) (all 1:1000). After secondary antibody incubation, bands were visualized using ECL (Epizyme, Shanghai, China) and quantified with ImageJ.

### ELISA Kit Assay

IL-25 and TGF-β levels were measured using ELISA kits (Cat. No. BMS6046, Thermo Scientific) (Cat. No. BMS608-4, Thermo Scientific) according to manufacturer instructions. Prior to measuring the TGF-β content in the cell supernatant, the supernatant was adjusted to approximately pH 3 using 1 M hydrochloric acid, incubated at room temperature for 10 min, and then neutralized to around pH 7 with 1 M sodium hydroxide solution before proceeding with ELISA detection.

### LDH Kit Assay

The LDH assay kit (Cat. No. A020-1–2, Jiancheng Bioengineering Institute, China) was used to measure the LDH level in the cell supernatant.

### METs Content Detection

The stimulated cell culture medium was collected and centrifuged at 1000 × g for 5 min. The supernatant was then collected, and the concentration was measured using a dsDNA assay kit (Cat. No. 12642ES60, Yeasen Biotechnology, Shanghai, China).

### METosis Formation Observation

METs release was visualized using fluorescence microscopy after SYTOX Green staining (0.25 µM, 10 min) (Cat. No. S7020, Thermo Scientific). Nuclei were counterstained with Hoechst 33,258 (biosharp).

### Fluorescent Staining of Lysosomes & Mitochondrial

Cells were stained with LysoTracker Red (Cat. No. L7528, Thermo Scientific) and MitoTracker Green FM (Cat. No. M46750, Thermo Scientific) at 37 ◦C for 60 min. The staining solution was removed, washed three times with PBS, and then observed under a fluorescence microscope.

### Detection of ROS

Cells were incubated with DCFH-DA (Cat. No. S0033S, Beyotime) for 60 min, washed, and observed under a fluorescence microscope.

### SEM and TEM Analysis

For SEM: RAW cells were seeded in a 6-well plate on sterile, 12 mm circular glass coverslips at a density of 2 × 10^5 cells/well and allowed to adhere overnight. Following the experimental treatments, the cells were fixed in situ with 2.5% glutaraldehyde in 0.1 M sodium cacodylate buffer, pH 7.4 for 1–2 h at 4 °C. The coverslips were then carefully removed from the plate and rinsed three times in cacodylate buffer. Post-fixation was performed with 1% osmium tetroxide in the same buffer for 1 h at 4 °C. Subsequently, the samples were dehydrated through a graded ethanol series (30%, 50%, 70%, 90%, 100%; 10 min each). Critical point drying was carried out using a Tousimis Autosamdri-815 Series A critical point dryer with liquid CO₂ to preserve delicate cellular structures. The dried samples were mounted onto aluminum stubs using double-sided conductive carbon tape and sputter-coated with a ~ 10 nm layer of gold–palladium using a Quorum Q150R S sputter coater to ensure conductivity. The samples were imaged using a Zeiss Sigma 300 VP field-emission scanning electron microscope operating at an accelerating voltage of 5 kV. Images were acquired using the secondary electron detector to maximize topographical contrast.

For TEM: RAW cells were cultured directly in the 6-well plate at a density of 5 × 10^5 cells/well. After treatment, the cells were fixed in the wells with 2.5% glutaraldehyde in 0.1 M sodium cacodylate buffer for 1 h at 4 °C. The cells were then gently scraped, transferred to a microcentrifuge tube, and pelleted by centrifugation at 1000 × g for 5 min. The resulting cell pellet was carefully handled to maintain its integrity. The pellet was post-fixed with 1% osmium tetroxide for 1 h at 4 °C, followed by en bloc staining with 1% aqueous uranyl acetate for 30 min. Dehydration was performed through a graded acetone series (50%, 70%, 90%, 100%; 10 min each). The pellet was infiltrated and embedded in EPON/Araldite resin according to the manufacturer's instructions. Ultrathin Sects. (70–90 nm thick) were cut using a Leica UC7 ultramicrotome with a diamond knife. The sections were collected on 200-mesh copper grids and post-stained with Reynolds' lead citrate and uranyl acetate. The grids were examined using a JEOL JEM-1400 Flash transmission electron microscope operating at an accelerating voltage of 120 kV. Digital images were captured using an integrated Gatan Olympus Morada CCD camera. Bright-field imaging mode was used to assess cellular ultrastructure.

### RNA Extraction and RT-qPCR

Total RNA was extracted using TRIzol reagent (Cat. No. AG21102, AGbio, China). cDNA was synthesized from 1 µg RNA. qPCR was performed using SYBR Green Master Mix (Cat. No. AG11701, AGbio, China). Relative expression was calculated via the 2^–ΔΔCt method [28]. Quantitative real-time PCR (qPCR) data were analyzed using the comparative threshold cycle (2 − ΔΔCt) method. The threshold cycle (Ct) values for both target genes and the endogenous reference gene were determined. The ΔCt value was calculated for each sample as: ΔCt = Ct (target gene) – Ct (reference gene). The ΔΔCt value was then determined by: ΔΔCt = ΔCt (test sample) – ΔCt (control sample). Finally, the relative expression level of the target gene was calculated as 2 − ΔΔCt. Data are presented as the mean fold change relative to the control group. Primers are listed in Table 1.

**Table 1:** Primer sequences used in this study

Gene name	Forward primer	Reverse primer	NCBI reference sequence
*Il1α*	TCTA TGAT GCAA GCTA TGGC TCA	CGGC TCTC CTTG AAGG TGA	NM_010554.4
*Il1β*	GAAA TGCC ACCT TTTG ACAG TG	TGGA TGCT CTCA TCAG GACA G	NM_008361.4
*Il3*	GGGA TACC CACC GTTT AACC A	AGGT TTAC TCTC CGAA AGCT CTT	NM_010556.4
*Il4*	GGTC TCAA CCCC CAGC TAGT	GCCG ATGA TCTC TCTC AAGT GAT	NM_021283.2
*Il5*	GCAA TGAG ACGA TGAG GCTT C	GCCC CTGA AAGA TTTC TCCA ATG	NM_010558.1
*Il6*	CTGC AAGA GACT TCCA TCCA G	AGTG GTAT AGAC AGGT CTGT TGG	NM_001314054.1
*Il7*	TTCC TCCA CTGA TCCT TGTT CT	AGCA GCTT CCTT TGTA TCAT CAC	NM_001313888.1
*Il9*	ATGT TGGT GACA TACA TCCT TGC	TGAC GGTG GATC ATCC TTCA G	NM_008373.2
*Il10*	CTTA CTGA CTGG CATG AGGA TCA	GCAG CTCT AGGA GCAT GTGG	NM_010548.2
*Il11*	GCGC TGTT CTCC TAAC CCG	GAGT CCAG ACTG TGAT CTCC G	NM_001290423.2
*Il12α*	CAAT CACG CTAC CTCC TCTT TT	CAGC AGTG CAGG AATA ATGT TTC	NM_001159424.3
*Il13*	TGAG CAAC ATCA CACA AGAC C	GGCC TTGC GGTT ACAG AGG	NM_008355.3
*Il14*	TCCT GAGT ACAT ACTG TGTG GAC	GCTG CATA GGTT CGGG ACTT C	NM_001005506.3
*Il15*	CATC CATC TCGT GCTA CTTG TG	GCCT CTGT TTTA GGGA GACC T	NM_001254747.2
*Il16*	AAGA GCCG GAAA TCCA CGAA A	GTGC GAGG TCTG GGAT ATTG C	NM_001360087.2
*Il17A*	TCAG CGTG TCCA AACA CTGA G	CGCC AAGG GAGT TAAA GACT T	NM_010552.3
*Il17F*	TGCT ACTG TTGA TGTT GGGA C	CAGA AATG CCCT GGTT TTGG T	NM_145856.2
*Il18*	GTGA ACCC CAGA CCAG ACTG	CCTG GAAC ACGT TTCT GAAA GA	NM_001357221.1
*Il19*	CTCC TGGG CATG ACGT TGAT T	GCAT GGCT CTCT TGAT CTCG T	NM_001009940.2
*Il20*	GTCT TGCC TTTG GACT GTTC T	AGGT TTGC AGTA ATCA CACA GC	NM_001311091.1
*Il21*	GGAC CCTT GTCT GTCT GGTA G	TGTG GAGC TGAT AGAA GTTC AGG	NM_001291041.1
*Il22*	ATGA GTTT TTCC CTTA TGGG GAC	GCTG GAAG TTGG ACAC CTCA A	NM_016971.2
*Il23*	CAGC AGCT CTCT CGGA ATCT C	TGGA TACG GGGC ACAT TATT TTT	NM_031252.2
*Il24*	GAGC CTGC CCAA CTTT TTGT G	TGTG TTGA AGAA AGGG CCAG T	NM_053095.3
*Il25*	ACAG GGAC TTGA ATCG GGTC	TGGT AAAG TGGG ACGG AGTT G	NM_080729.3
*Il27*	CTGT TGCT GCTA CCCT TGCT T	CTCC TGGC AATC GAGA TTCA G	NM_145636.2
*Il28B*	CTCC TGGC AATC GAGA TTCA G	GTGG GAAC TGCA CCTC ATGT	NM_177396.1
*Il31*	TCAG CAGA CGAA TCAA TACA GC	TCGC TCAA CACT TTGA CTTT CT	NM_001431110.1
*Il33*	ATTT CCCC GGCA AAGT TCAG	AACG GAGT CTCA TGCA GTAG A	NM_001164724.2
*Il34*	TTGC TGTA AACA AAGC CCCA T	CCGA GACA AAGG GTAC ACAT TT	NM_001135100.2
*Il40*	ACTG GAAG TTTA TCCC CAAA GC	CGGA GTCA TGCA CAAC CTTT TT	NM_029964.1
*Acta2*	TGCT GGAC TCTG GAGA TGGA	GCCA TGTT CTAT CGGGTACTTC	NM_007392.3
*Col1a1*	CTCC GTGG TCTC TGAG GAGA	GGGA TGTT GTCG AGGG AGTA	NM_007742.4
*Tnf-α*	CCCT CACA CTCA GATC ATCT TCT	GCTA CGAC GTGG GCTA CAG	NM_001278601.1
*Tgf-β*	GTGT GGAG CAAC ATGT GGAA CTCT	CGCT GAAT CGAA AGCC CTGT A	NM_011577.2
*Actb*	GGCT GTAT TCCC CTCC ATCG	CCAG TTGG TAAC AATG CCAT GT	NM_007393.5
*Gapdh*	AGGT CGGT GTGA ACGG ATTT G	TGTA GACC ATGT AGTT GAGG TCA	NM_001289726.2

### Statistical Analysis

At least three separate experiments were performed, and the results are expressed as the mean ± standard error of the mean. Two-tailed Student’s t-test was used to analyze the statistical significance of the differences between two groups. One-way analysis of variance was used to assess the statistical significance of the differences among multiple groups. Statistical significance was set at p < 0.05. GraphPad Prism 5.0 (San Diego, CA, USA) was used for all analyses.

## Results

### CCl₄-Induced Liver Fibrosis Demonstrate Significantly Augmented METs Generation

**Fig. 1:**
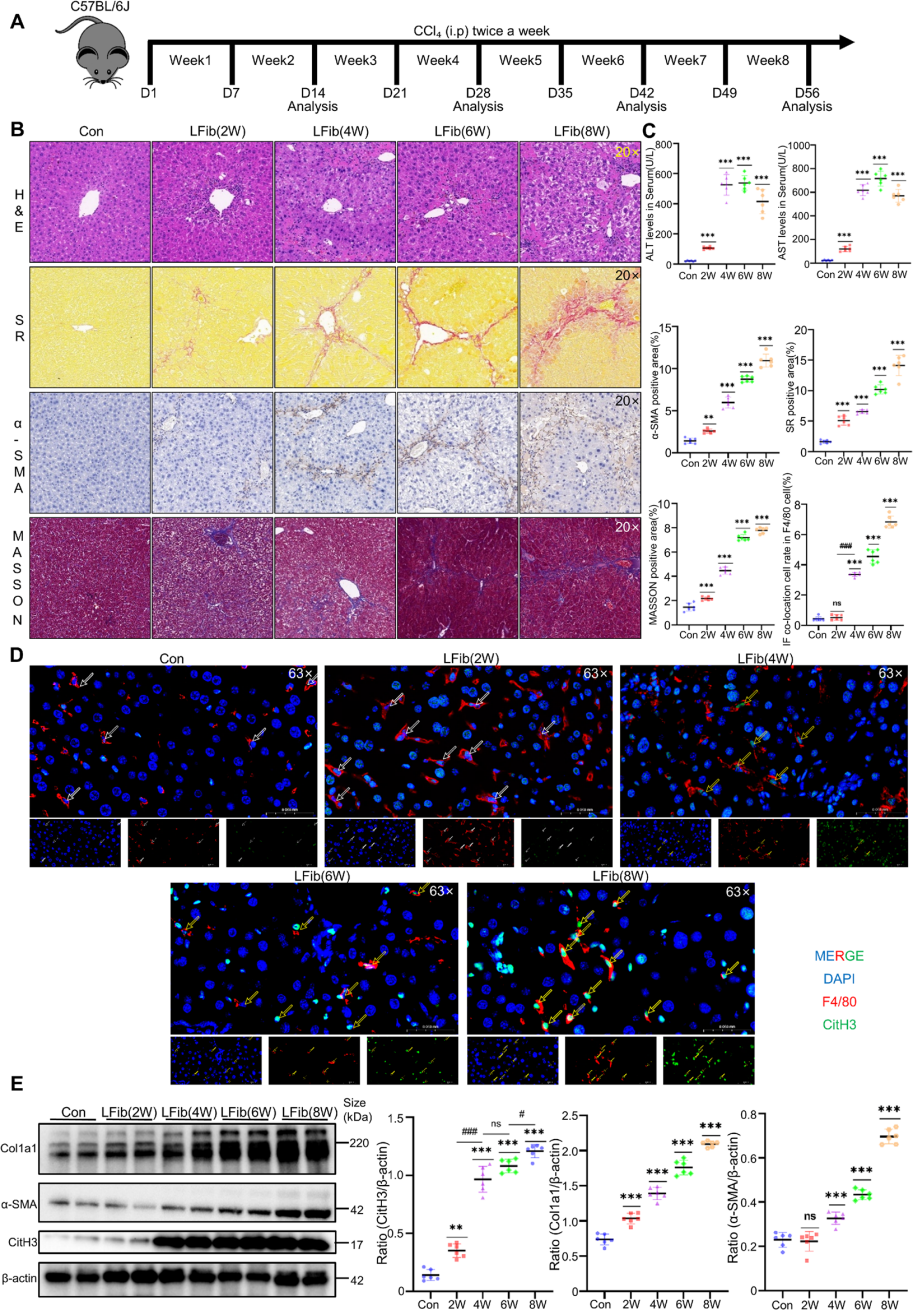
CCl₄-induced fibrotic murine livers demonstrate significantly augmented METs generation. **A** Flowchart of liver fibrosis modeling in C57 mice (*n* = 10 per group). **B** Representative histological staining of liver lobular tissues in a mouse model of liver fibrosis, including H&E, Sirius Red, Masson's trichrome, and α-SMA IHC staining with the semi-quantitative results of the processed images from each group (*n* = 6 per group). **C** Measurement of ALT and AST levels in mouse peripheral blood serum (*n* = 6 per group). **D** Double immunofluorescence staining of mouse liver tissue sections using F480 and CitH3 antibodies, with DAPI for nuclear counterstaining with the semi-quantitative results of the processed images from each group, yellow arrows indicate METs (F4/80⁺CitH3⁺), and white arrows indicate macrophages (F4/80⁺CitH3⁻). (*n* = 6 per group). **(E)** WB analysis of CitH3, Col1a1 and α-SMA expression in liver tissues with the semi-quantitative results from the grayscale analysis of protein bands for each group, representative western blots from three independent experiments are shown; each lane represents an individual mouse. (*n* = 6 per group). Mean ± SEM; *p < 0.05, **p < 0.01, ***p < 0.001, ****p < 0.0001. Student’s t-test

METs formation was investigated in a CCl₄-induced liver fibrosis model. Fibrosis was induced by i.p. CCl₄ injection twice weekly for 2–8 weeks (Fig. 1A). Histopathological analysis revealed progressive inflammatory infiltrates and collagen deposition, with severity increasing over time (Fig. 1B). Serum ALT and AST levels were elevated at 2, 4, and 6 weeks but declined at 8 weeks, likely due to reduced hepatocyte mass (Fig. 1C). The elevated expression of α-SMA and Col1a1 in liver tissue also confirmed the formation of fibrosis (Fig. 1E). Immunofluorescence double staining showed increased citrullinated histone H3 (CitH3, a MET marker) co-localized with F4/80-positive macrophages in fibrotic livers (Fig. 1D). The elevated expression of CitH3 in liver tissue also confirmed it (Fig. 1E), indicating MET formation as a pathological feature of experimental liver fibrosis.

### Activation of HSCs by METs through the CCDC25 Pathway Exacerbates Liver Fibrosis

**Fig. 2:**
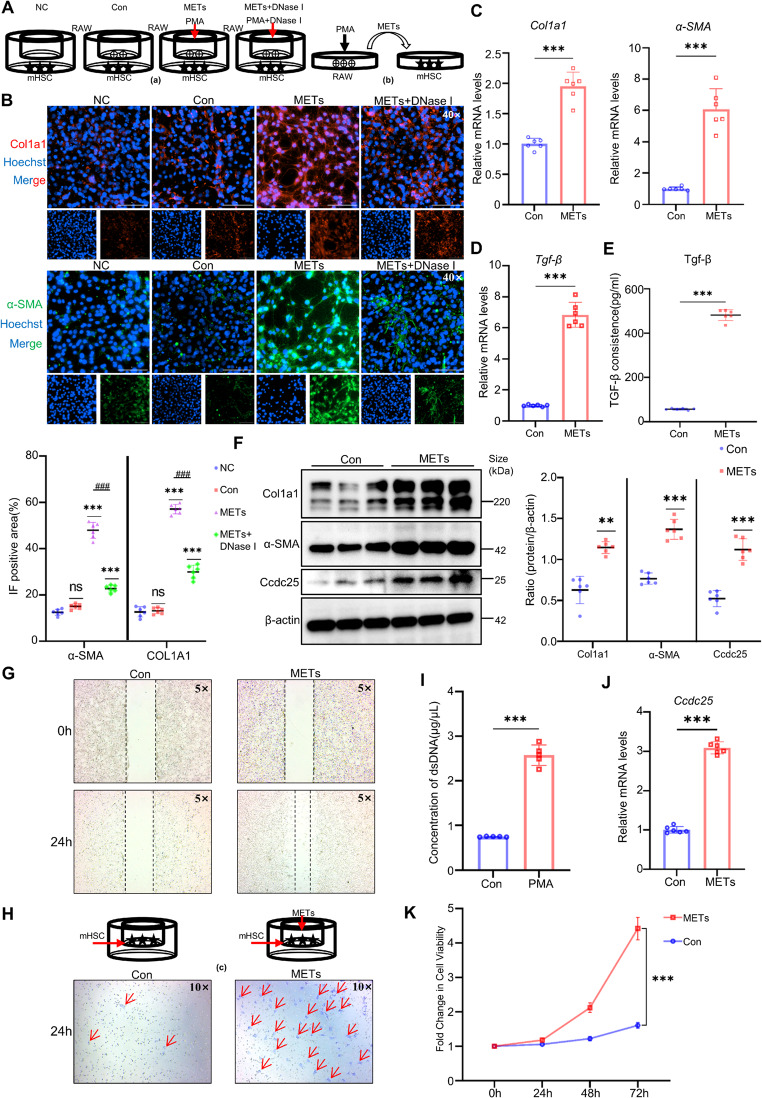
Activation of HSCs by METs exacerbates hepatic fibrogenesis. **A, H** The three co-culture methods used (*n* = 6 per group). **B** The IF staining of Col1a1 and α-SMA in mHSC cells after 48 h of METs (2 μg/mL) treatment with the semi-quantitative results of the processed images from each group (*n* = 6 per group). **C** qPCR analysis of Col1a1, α-SMA expression in mHSC cells after 48 h of METs treatment (*n* = 6 per group). **D** qPCR analysis of Tgf-β expression in mHSC cells after 48 h of METs treatment (*n* = 6 per group). **E** the level of Tgf-β in the mHSC cell supernatant after 48 h of METs treatment (*n* = 6 per group). **F** WB analysis of Ccdc25, Col1a1 and α-SMA expression in mHSC cells after 48 h of METs treatment with the semi-quantitative results from the grayscale analysis of protein bands for each group, representative western blots from three independent experiments are shown, each lane represents a biological replicate, and a subset of samples is displayed for clarity (*n* = 6 per group). **G** The wound healing assay of mHSC cells (*n* = 6 per group). **H** Migration assay of mHSC cells, red arrows indicate activated mHSCs that have migrated through the microporous membrane to the lower chamber (*n* = 6 per group). **I** Measurement of dsDNA content in the supernatant of RAW cells stimulated with PMA (*n* = 5 per group). **J** qPCR analysis of Ccdc25 expression in mHSC cells after 48 h of METs treatment (*n* = 6 per group). **K** CCK-8 assay was used to detect the proliferation of mHSC cells stimulated with METs, for 0 h,24 h,48 h, and 72 h. (*n* = 6 per group). Mean ± SEM; *p < 0.05, **p < 0.01, ***p < 0.001, ****p < 0.0001. Student’s t-test

**Fig. 3:**
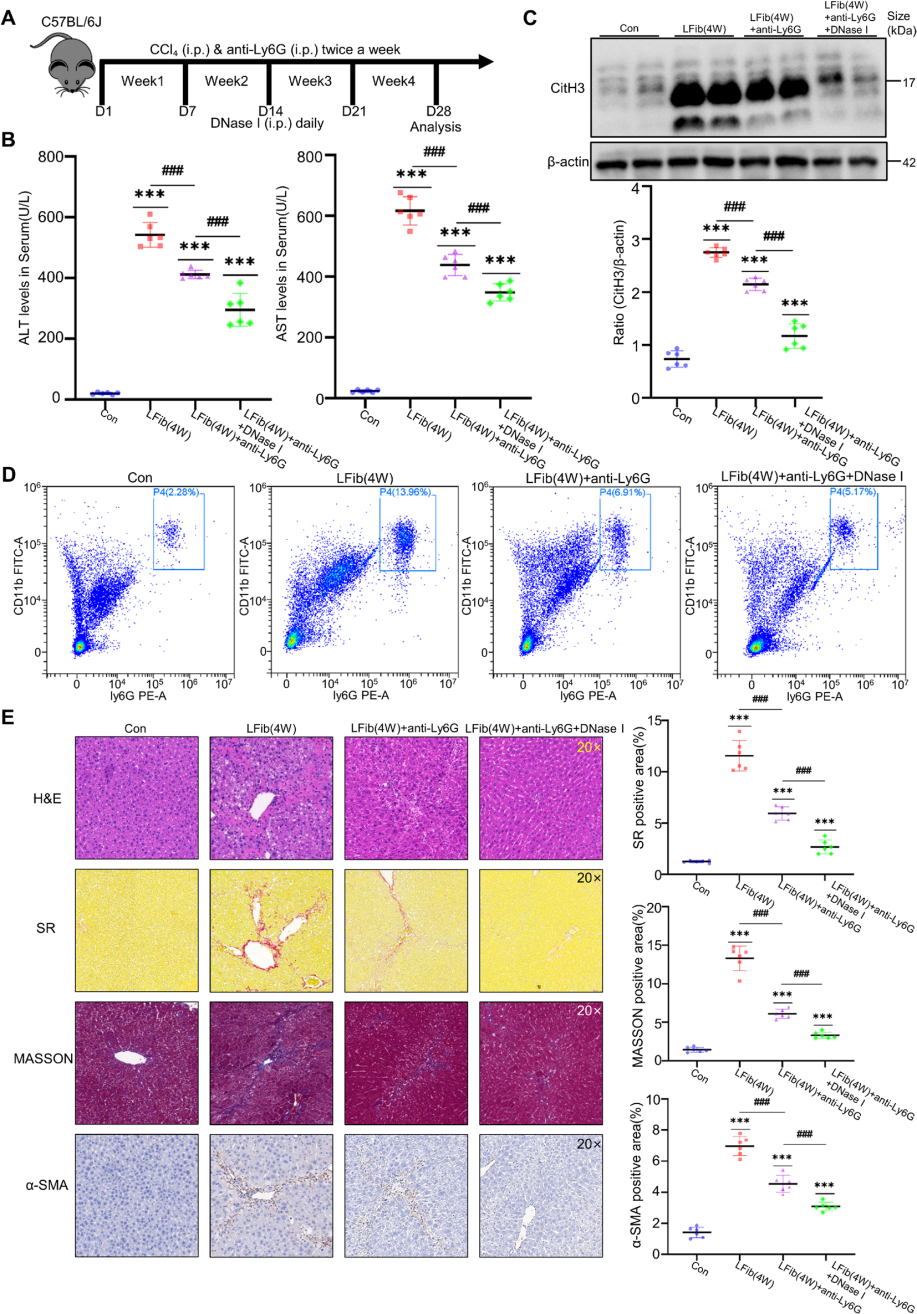
Administration of DNase I to mice can slow the progression of liver fibrosis.** A** Flowchart of liver fibrosis modeling with anti-Ly6G and Dnase I treatment in C57 mice (*n* = 10 per group). **B** Measurement of ALT and AST levels in mouse peripheral blood serum (*n* = 6 per group). **C** WB analysis of CitH3 expression in liver tissues with the semi-quantitative results from the grayscale analysis of protein bands for each group, representative western blots from three independent experiments are shown, each lane represents a biological replicate, and a subset of samples is displayed for clarity (*n* = 6 per group). **D** Flow cytometry analysis of immune cell populations in mouse liver tissue (*n* = 6 per group). **E** Histological staining of liver tissues in a mouse model, including H&E, Sirius Red, Masson's trichrome, and α-SMA IHC staining with the semi-quantitative results of the processed images from each group (*n* = 6 per group). Mean ± SEM; *p < 0.05, **p < 0.01, ***p < 0.001, ****p < 0.0001. Student’s t-test

We categorized the in vitro co-culture system into three distinct approaches: direct co-culture with RAW cells seeded in the upper layer and mHSC cells in the lower layer (a); culture of mHSC cells using conditioned medium containing METs derived from PMA-stimulated RAW cells (b); and stimulation of mHSC cells in the upper chamber with lower chamber medium containing METs to examine mHSC cell migration (c) (Fig. 2 A, H, I). Fluorescence staining of the co-culture using method (a) revealed that in the absence of PMA stimulation, co-culturing RAW cells with mHSC cells slightly increased the expression of type I collagen and α-SMA, though the difference was not statistically significant. After PMA stimulation to induce METs production, the protein expression of type I collagen and α-SMA was significantly elevated. In contrast, the use of the METs inhibitor DNase I led to a marked reduction in the expression of these proteins (Fig. 2B). The results from the co-culture method (b) showed that under METs stimulation, the protein and RNA expression of type I collagen and α-SMA in mHSC cells were significantly increased (Fig. 2C), as was the RNA expression of TGF-β (Fig. 2D). Additionally, the TGF-β content in the co-culture supernatant was significantly elevated (Fig. 2E). The CCK-8 assay for detecting mHSC proliferation indicated that mHSC proliferation increased significantly over time (Fig. 2K), and the wound healing assay demonstrated that METs stimulation markedly enhanced mHSC cell proliferation (Fig. 2G). The cell migration assay results from method (c) revealed that mHSC cells stimulated by METs exhibited a significantly increased migration rate (Fig. 2H). Furthermore, the protein and RNA expression of CCDC25 in mHSC cells cultured by method (b) was significantly elevated, suggesting that METs may activate and promote the proliferation of hepatic stellate cells via CCDC25 (Fig. 2F, J). In vivo, we established a mouse model of liver fibrosis with neutrophil depletion. Given that macrophages become the dominant players in the immune response after neutrophil depletion, owing to their substantial presence in the liver, we hypothesized that METs play a significant role in this model. Therefore, we used DNase I to deplete METs and observed changes in the degree of liver fibrosis (Fig. 3A). Furthermore, since Fig. 1D shows that METs are already significantly generated by 4 weeks, we selected mice with 4-week fibrosis as the study subjects. Flow cytometry analysis of immune cells in mouse liver tissue confirmed significant depletion of neutrophils (Fig. 3D). Serum ALT and AST results indicated that liver injury was markedly reduced in the neutrophil‑depleted liver fibrosis group, and further decreased in the neutrophil‑depleted + DNase I liver fibrosis group compared to the neutrophil‑depleted group alone (Fig. 3B), a finding supported by liver histopathological and IHC staining (Fig. 3E). Moreover, the expression of Cith3 in liver tissue showed a consistent trend, demonstrating that DNase I treatment after neutrophil depletion also significantly reduced Cith3 expression (Fig. 3C). In addition, triple immunofluorescence staining of liver tissue sections for F4/80, MPO, and CitH3 was performed, further confirming the presence of METs during the progression of liver fibrosis and demonstrating that, following neutrophil depletion, the remaining ETs in the liver primarily originate from macrophages as METs (Supplementary Fig. 1 A), suggesting the production of METs and their important role in fibrosis.

### Upregulated Hepatocyte-derived IL-25 Promotes METs Formation in Liver Fibrosis

**Fig. 4:**
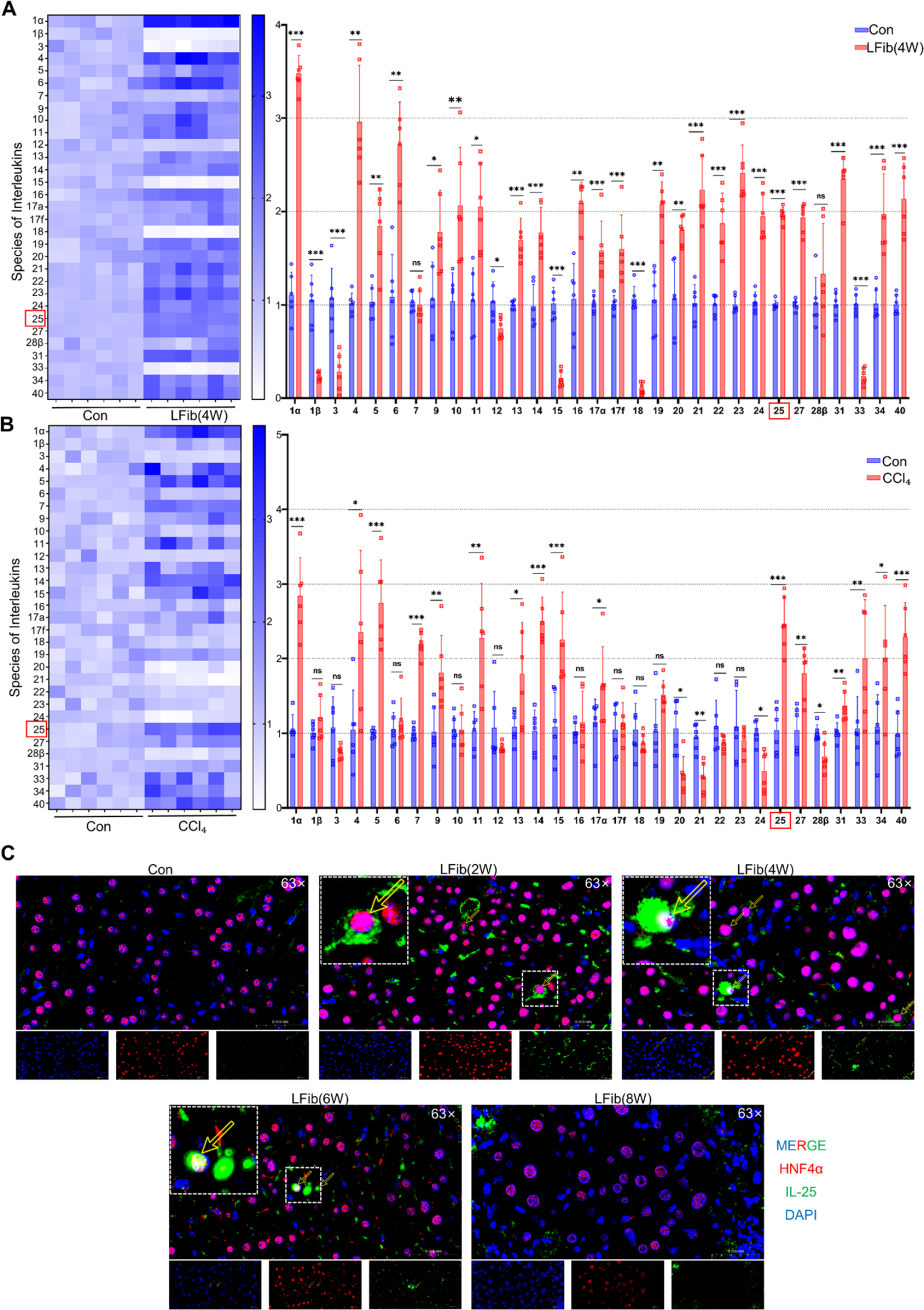
Upregulated hepatocyte-derived IL-25 promotes the formation of METs in liver fibrosis. **A** qPCR analysis of 31 kinds of Interleukins expression in 4 W LFib mice liver tissues, The heatmap and bar graph visually represent the fold changes of IL-25 and other interleukins. (*n* = 6 per group). **B** qPCR analysis of 31 kinds of Interleukins expression in AML-12, stimulated with culture medium containing 0.2% CCl₄ for 12 h. The heatmap and bar graph visually represent the fold changes of IL-25 and other interleukins. (*n* = 6 per group). **C** Double immunofluorescence staining of mouse liver tissue sections using HNF4α and Il-25 antibodies, with DAPI for nuclear counterstaining with the semi-quantitative results of the processed images from each group, yellow arrows indicate hepatocyte-derived IL-25 (HNF4α⁺Il-25⁺). (*n* = 6 per group). Mean ± SEM; *p < 0.05, **p < 0.01, ***p < 0.001, ****p < 0.0001. Student’s t-test

During the progression of liver fibrosis, damaged hepatocytes release intercellular communication factors, among which interleukins represent a crucial category. The 4 W LFib model induced by intraperitoneal injection of CCl₄ is well-established and widely recognized. Furthermore, our staining results demonstrated evident METs formation in mice with LFib at this time point. Therefore, we selected mice with 4 W LFib as the subjects for interleukin screening. Interleukin screen in 4 W fibrotic liver tissues identified IL-25 as significantly upregulated (Fig. 4A). An in vitro model was established to simulate fibrotic progression in mice. Initially, the immortalized mouse hepatocyte line AML-12 was stimulated with CCl₄. Cells were treated with 0.2% CCl₄ in culture medium for 12, 24, 48 h (Supplementary Fig. 2 A). The results showed that hepatocytes exhibited no significant apoptosis at the 12-h stimulation time point, while the level of the injury marker LDH was markedly elevated. Thus, 12 h was selected as the duration for in vitro simulation to screen for interleukins. In vitro, CCl₄ treatment of AML-12 hepatocytes for 12 h and induced IL-25 mRNA significantly upregulated (Fig. 4B) with injury (Supplementary Fig. 2B) and secretion in the cell supernatant (Supplementary Fig. 2D). Immunofluorescence confirmed IL-25 co-localization with HNF-4α-positive hepatocytes in 4 W fibrotic livers (Fig. 4C). Systemic IL-25 levels were elevated in serum and liver tissue (Supplementary Fig. 2 C, E). To further investigate the relationship between IL-25 and METs formation across various stages of liver fibrosis, we performed immunofluorescence double staining on the aforementioned liver tissues (2w,4w,6w,8w). The staining results revealed significant colocalization of IL-25 with HNF4α in mice at the 2-week modeling time point compared to the control group. Furthermore, IL-25 expression exhibited a marked increase as modeling duration extended. Though, the expression was markedly reduced in the 8‑week model, which may be attributed to the substantial loss of hepatocytes, alterations in the immune microenvironment during late‑stage liver fibrosis, as well as the negative feedback regulation by downstream inflammatory factors (Supplementary Fig. 2 C, E). Interestingly, colocalization of CitH3 with F4/80 was not yet prominent in 2-week model mice compared to controls, despite showing early macrophage aggregation and activation. In contrast, 4-week fibrotic mice demonstrated clear colocalization of CitH3 and F4/80 (Fig. 1D-E). This colocalization, along with CitH3 expression, increased progressively with longer modeling time, manifested as macrophage deformation and a continuous, substantial elevation in CitH3 levels. These findings collectively indicate that early IL-25 expression preceded METs formation, suggesting hepatocyte-derived IL-25 may as an initiator of METosis.

### IL-25 Promotes the Formation of METs In Vitro

**Fig. 5:**
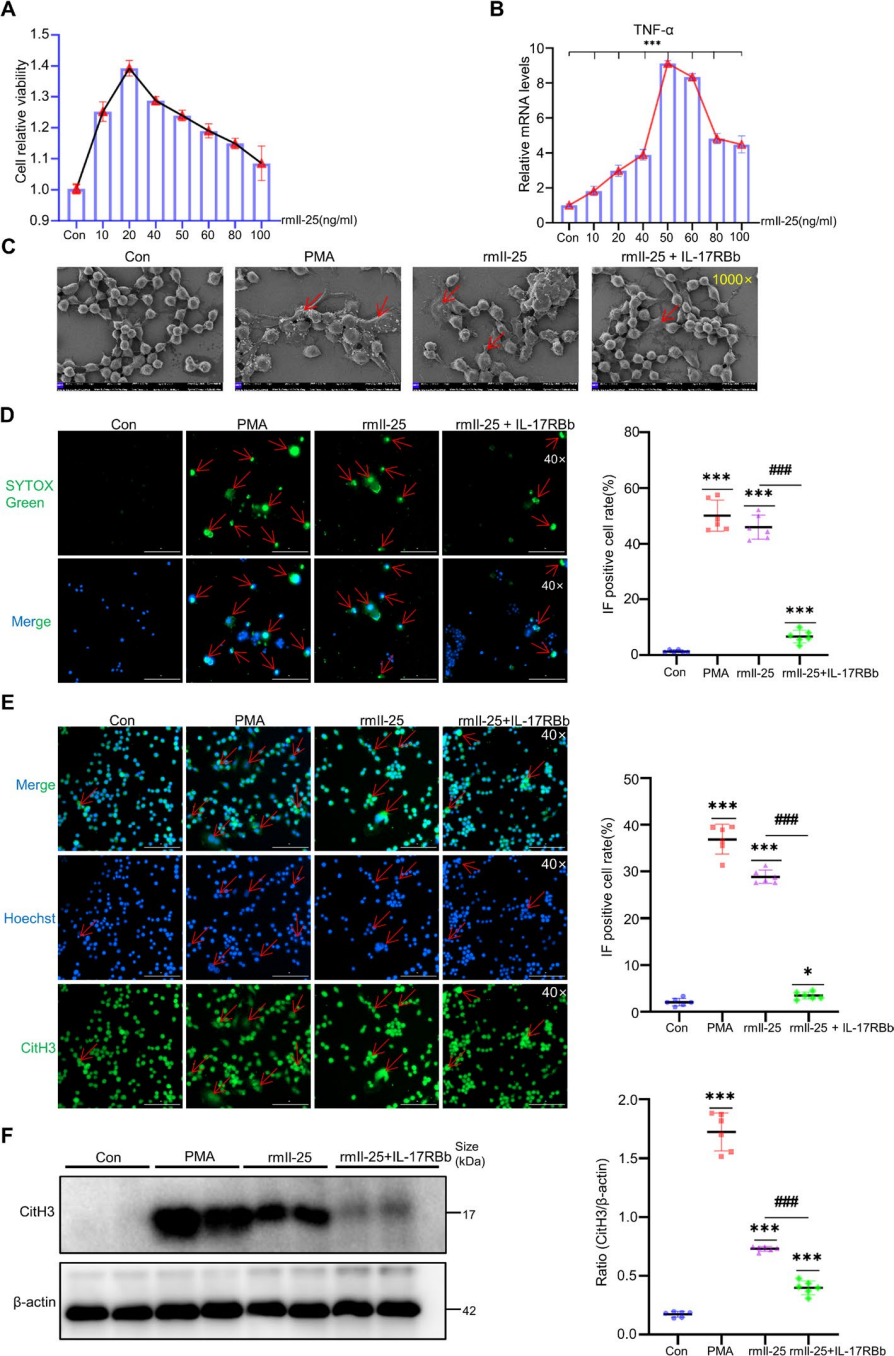
IL-25 promotes the formation of METs in vitro.** A** CCK-8 assay was used to detect the proliferation of RAW cells stimulated with IL-25, as indicated by relative cell viability (*n* = 6 per group). **B** qPCR analysis of Tnf-α expression in RAW cells stimulated with Il-25 for 24 h (*n* = 6 per group). **C** Observation of METs formation in RAW cells under scanning electron microscopy, red arrows indicate METs formation. (*n* = 4 per group). **D** Observation of METs formation in RAW cells by SYTOX Green staining, with Hoechst for nuclear counterstaining with the semi-quantitative results of the processed images from each group, red arrows indicate METs formation. (*n* = 6 per group). **E** Observation of METs formation in RAW cells by CitH3 IF staining, with Hoechst for nuclear counterstaining with the semi-quantitative results of the processed images from each group, CitH3-positive structures were defined as those with perinuclear staining co-localized with Hoechst, red arrows indicate extracellular fibrous CitH3⁺ structures with characteristic chromatin decondensation (*n* = 6 per group). **F** WB analysis of CitH3 expression in RAW cells with the semi-quantitative results from the grayscale analysis of protein bands for each group, representative western blots from three independent experiments are shown, each lane represents a biological replicate, and a subset of samples is displayed for clarity (*n* = 6 per group). For IL-17RB blockade, cells were pre-incubated with IL-17RBb 50 ng/ml for 1 h prior to stimulation with IL-25. The inhibitor was present throughout the subsequent stimulation period. Mean ± SEM; *p < 0.05, **p < 0.01, ***p < 0.001, ****p < 0.0001. Student’s t-test

PMA was employed as a positive control while utilizing recombinant IL-25 to stimulate macrophages for functional validation. Initially, the optimal concentration of IL-25 for macrophage treatment was screened. Based on prior literature, macrophages were exposed to a gradient of IL-25 concentrations (10, 20, 40, 50, 60, 80, and 100 ng/ml) for 24 h. CCK-8 assay results demonstrated no significant cytotoxicity of IL-25 toward macrophages (Fig. 5A). Following 24-h treatment, RNA was extracted for qPCR analysis. The qPCR data revealed peak mRNA expression of TNF-α (secretory protein markers of macrophage activation) at 50 ng/ml IL-25(Fig. 5B). Consequently, 50 ng/ml was selected as the optimal concentration for subsequent IL-25 stimulation. IL-25 treatment (50 ng/ml) induced MET formation in macrophages, similar to PMA stimulation, as shown by SEM, Sytox Green staining, and CitH3 expression, this effect was blocked by IL-17RB inhibition (Fig. 5C-F). Additionally, we employed primary mouse bone marrow-derived macrophages (BMDMs) for further investigation, which provided additional evidence that IL-25 induces the formation of METs (Supplementary Fig. 2 F). These collective data confirm that IL-25 specifically promotes IL-17RB-mediated METs formation, an effect effectively abrogated by receptor blockade.

### Hepatocyte-derived IL-25 Promotes Liver Fibrosis Progression

**Fig. 6:**
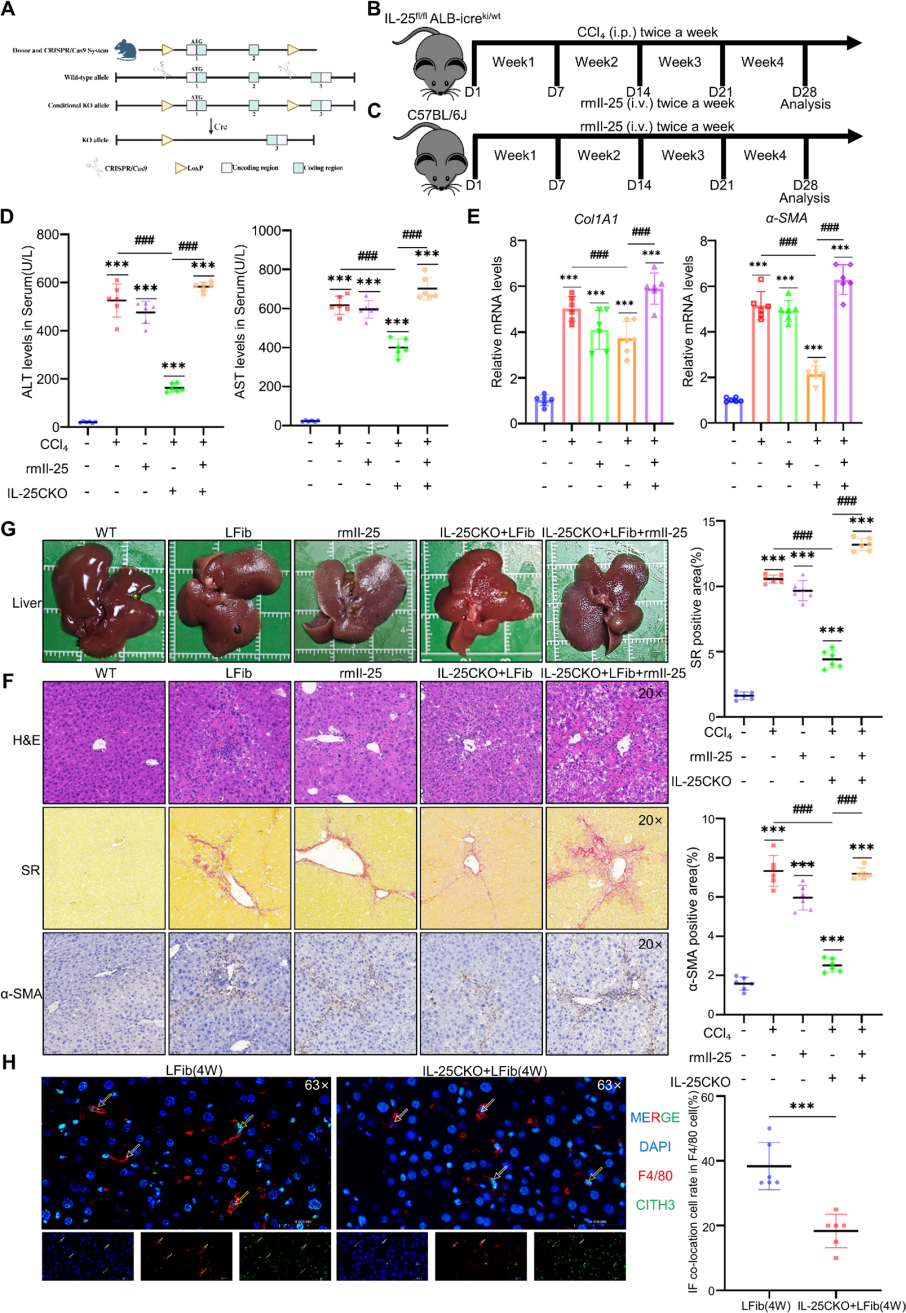
Hepatocyte-derived IL-25 promote the progression of liver fibrosis. **A** IL-25CKO mice create strategy. **B** Flowchart of liver fibrosis modeling in IL-25^ fl/fl^ ALB-icre^ki/wt^ mice (*n* = 10 per group). **C** Flowchart of liver fibrosis modeling induced by IL-25 treatment in C57 mice (*n* = 10 per group). **D** Measurement of ALT and AST levels in mouse peripheral blood serum (*n* = 6 per group). **E** qPCR analysis of Col1a1 and α-SMA expression in mice liver tissues (*n* = 6 per group). **F** Representative histological staining of liver tissues in a mouse model of liver fibrosis, including H&E, Sirius Red, and α-SMA IHC staining with the semi-quantitative results of the processed images from each group (*n* = 6 per group). **G** Mice liver entity photo (*n* = 6 per group). **H** Double immunofluorescence staining of mouse liver tissue sections using F4/80 and Cith3 antibodies, with DAPI for nuclear counterstaining with the semi-quantitative results of the processed images from each group (*n* = 6 per group). Mean ± SEM; *p < 0.05, **p < 0.01, ***p < 0.001, ****p < 0.0001. Student’s t-test

To elucidate the role of IL-25 in liver fibrosis, we generated hepatocyte-specific IL-25 knockout mice (IL-25CKO) (IL-25^ fl/fl^ ALB-icre^ki/wt^) (Fig. 6A) and subjected them to CCl₄ and (or) IL-25-induced liver fibrosis for 4 weeks using the established protocol (Fig. 6B). We also established an administration group in C57 mice that received intraperitoneal injections of mrIL-25 to verify the fibrogenic effect of IL-25 alone. (Fig. 6C). IL-25CKO mice showed attenuated liver fibrosis after CCl₄ induction, while IL-25 administration exacerbated liver fibrosis (Fig. 6D-G). Consistent with this, reduced MET formation was observed in IL-25CKO mice (Fig. 6H), confirming the pro-fibrotic role of hepatocyte-derived IL-25. Additionally, the results of serum IL-25 levels in both CKO mice and wild-type mice from the liver fibrosis and control groups demonstrated that IL-25 content was significantly reduced in CKO mice during liver fibrosis (Supplementary Fig. 2 J). The genotyping results of IL-25 CKO mice and the detection results of liver tissue protein and mRNA expression are shown in the figure (Supplementary Fig. 2G, H, I).

### IL-25 Binding to IL-25R on Macrophages Triggers ROS Burst via NOX Activation and Mitophagy, Inducing Lysosomal Activation and MET Formation

**Fig. 7:**
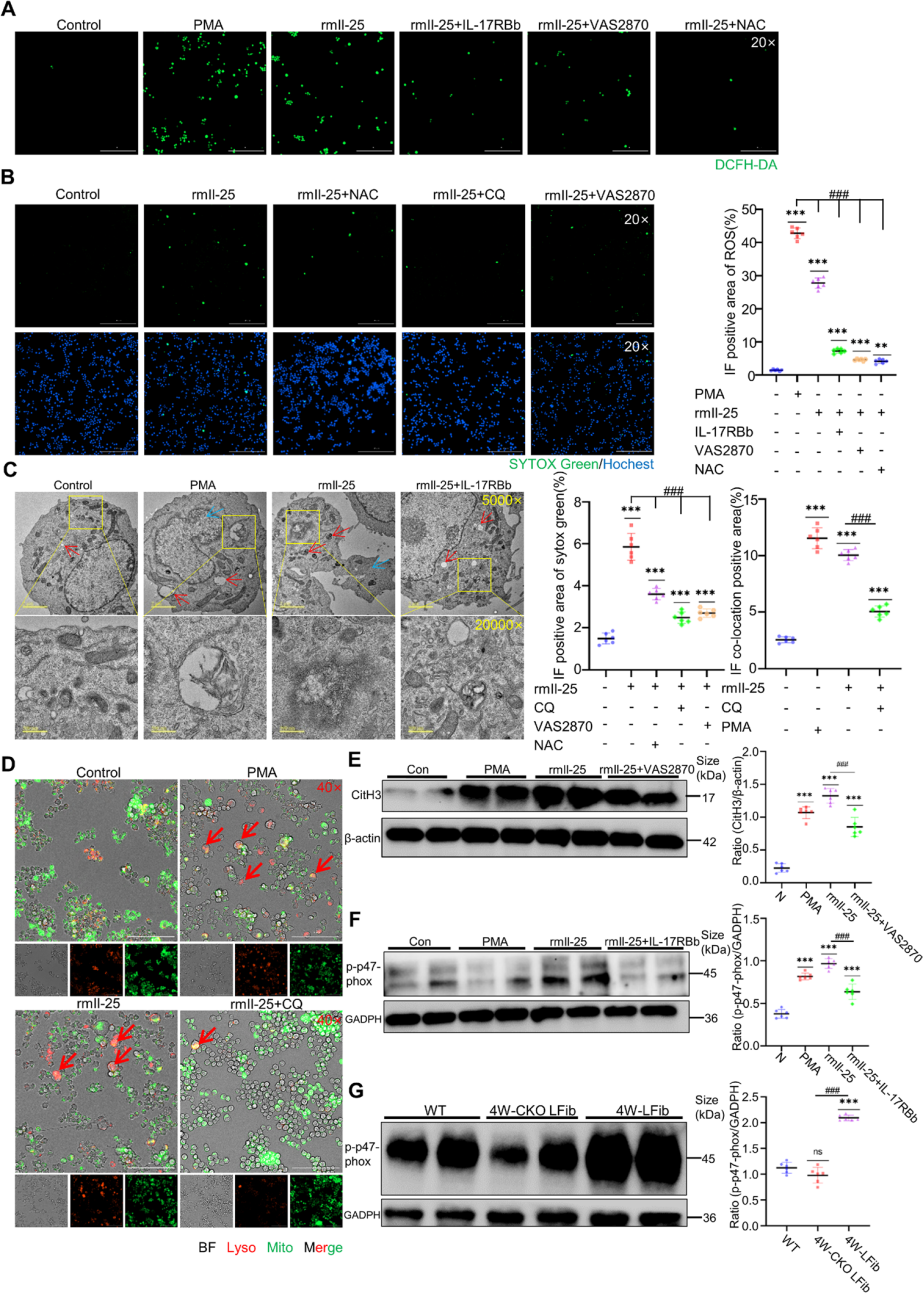
IL-25 to the IL-25 receptor on macrophages triggers ROS burst via NOX activation and mitophagy, consequently inducing lysosomal activation and MET formation. **A** Cell ROS assay of RAW264.7 by DCFH-DA with the semi-quantitative results of the processed images from each group (*n* = 6 per group). **B** Observation of METs formation in RAW cells by SYTOX Green staining, with Hochest for nuclear counterstaining with the semi-quantitative results of the processed images from each group (*n* = 6 per group). **C** Observation of lysosome and mitochondrial morphology in RAW cells under transmission electron microscopy, red arrows indicate activated lysosomes and autophagosome formation, and blue arrows indicate METs formation. (*n* = 4 per group). **D** Mitochondrial & Lysosomes staining in RAW cell with the semi-quantitative results of the processed images from each group (*n* = 6 per group). **E** WB analysis of CitH3 expression in RAW cells with the semi-quantitative results from the grayscale analysis of protein bands for each group, representative western blots from three independent experiments are shown, each lane represents a biological replicate, and a subset of samples is displayed for clarity (*n* = 6 per group). **F-G** WB analysis of phosphorylated p47phox protein expression in RAW cells and liver tissues with the semi-quantitative results from the grayscale analysis of protein bands for each group, representative western blots from three independent experiments are shown, each lane represents a biological replicate, and a subset of samples is displayed for clarity (*n* = 6 per group). Cells were pretreated with VAS2870 25 μM for 30 min prior to stimulation, NAC 10 μM for 1 h before stimulation, CQ 10 μM for 2 h before stimulation. Mean ± SEM; *p < 0.05, **p < 0.01, ***p < 0.001, ****p < 0.0001. Student’s t-test

Following 6-h exposure to PMA, macrophages demonstrated significant induction of METs, accompanied by an elevation in intracellular ROS production. This result was the same upon IL-25 treat (Fig. 7A). This observation reversed those obtained in the N-acetylcysteine (NAC, a ROS scavenger) and IL-17RBb treatment cohort, with the NAC-administered and IL-17RBb treatment group exhibiting a marked reduction in METs production (Fig. 7B,5D). Concurrently, PMA stimulation enhanced autophagosome formation within macrophages, with a pronounced increase in mitochondrial autophagy, ultrastructural validation by transmission electron microscopy (TEM) unequivocally confirmed these results (Fig. 7C). Macrophages stimulated with IL-25 and PMA exhibited significant activation of lysosomes and mitophagy after treatment, characterized by intracellular dispersion of lysosomes and their colocalization with mitochondria (Fig. 7D). Fluorescence staining analysis revealed that control macrophages displayed normal morphology, with mitochondria and lysosomes distributed separately within the cells, showing little or no colocalization. In contrast, macrophages in the PMA and IL-25 stimulated groups showed marked activation, manifested by a significant increase in cell volume. These activated cells exhibited pronounced colocalization of mitochondria and lysosomes within the cytoplasm. Furthermore, depending on the stimulation level, lysosomes displayed intracellular dispersion, accompanied by a reduction in mitochondrial content. Additionally, no detectable lysosomal or mitochondrial activity signals were observed within MET-enriched regions. This suggests that lysosomal activation impairment and mitophagy may participate in the formation of METs. The subsequent cellular rupture caused by MET formation leads to the release of cellular contents, thereby resulting in the disappearance of mitochondrial and lysosomal signals. To confirm the role of lysosomal in METs formation, chloroquine (CQ), a pharmacological inhibitor of lysosomal function, was employed. Experimental results demonstrated that CQ treatment alone not only significantly reduced activation of lysosomes but also effectively attenuated PMA-induced MET generation, suggesting a critical dependency on intact lysosomal pathways for MET biogenesis (Fig. 7B, D). To further investigate the mechanism by which IL-25 activates ROS burst, the NOX-specific inhibitor VAS2870 was employed. Our results demonstrated that pretreatment with VAS2870 significantly reduced MET generation and markedly decreased ROS levels within macrophages following IL-25 administration, compared to the IL-25-only group (Fig. 7A-B). Similarly, the VAS2870 + IL-25 group exhibited reduced CitH3 expression compared to IL-25 only (Fig. 7E). Macrophages treated solely with IL-25 showed elevated expression of phosphorylated p47phox protein, indicating NOX activation. This phenomenon, however, was not observed in the IL-25 + IL-17RBb group (Fig. 7F). This phenomenon was also observed in the liver tissues of the fibrotic mouse model. Compared with wild-type mice, IL-25 CKO mice with liver fibrosis exhibited a significantly reduced expression of phosphorylated p47phox (Fig. 7G). These findings collectively suggest that IL-25 induces ROS burst by activating NOX.

## Discussion

Liver fibrosis is a chronic global health issue that can progress to cirrhosis and hepatocellular carcinoma [29]. Its pathogenesis involves complex cellular mechanisms [30, 31]. Upon injury, macrophages are recruited and activated, releasing METs to neutralize threats [32, 33]. During the early inflammatory phase in the liver, circulating macrophages are recruited to the hepatic tissue [34]. These macrophages, activated by multiple cytokines, subsequently release METs to neutralize external threats. However, akin to NETs, METs function as a double-edged sword. Our findings demonstrate that PMA stimulation induces sustained inflammatory activation in macrophages, which subsequently triggers mitochondrial damage and a burst of ROS. This cascade leads to persistent mitophagy and lysosomal activation. Critically, such prolonged cellular stress culminates in autophagic dysregulation and lysosomal membrane destabilization, resulting in the release of extracellular traps and exacerbation of hepatic inflammation. Uncontrolled overproduction of METs exacerbates fibrotic progression rather than mitigating injury. This paradoxical effect underscores the critical need to regulate MET dynamics. In this study, we systematically investigate the dual role of METs in liver fibrosis and propose a therapeutic strategy to attenuate fibrosis by suppressing excessive MET formation during the initial inflammatory phase.

External stimuli typically induce excessive ROS generation in biological systems, with oxidative stress established as a key pathway driving cellular and tissue damage [35]. During liver fibrosis, persistently activated macrophages produce substantial ROS, which subsequently damages cellular organelles and triggers compensatory autophagy. Critically, ROS generation operates as a self-amplifying cascade: damaged organelles (e.g., mitochondria) further release ROS in a phenomenon termed "respiratory burst," exacerbating injury [36]. While uncontrolled ROS accumulation causes mitochondrial dysfunction and cell death, lysosomal activation and autophagosome-lysosome fusion serve as protective mechanisms by degrading damaged mitochondria to halt ROS overload [37]. However, chronic lysosomal activation—particularly evident in fibrotic diseases—paradoxically increases lysosomal membrane permeability (LMP) and promotes rupture [38, 39]. Our experimental validation using macrophages co-treated with PMA and the ROS scavenger NAC demonstrated significantly reduced LMP. This finding directly supports our hypothesis that ROS-driven lysosomal destabilization is a critical checkpoint in macrophage dysfunction during fibrosis.

Extracellular traps (ETs) were initially defined as DNA-based structures decorated with antimicrobial proteins, primarily released by neutrophils. Although their formation mechanisms remain incompletely elucidated, key regulatory events have been identified [40, 41]. Notably, inflammatory stimuli activate macrophages to generate ROS, which represent a critical driver of METs formation. Excessive ROS production triggers lysosomal rupture, leading to calcium influx and subsequent histone citrullination—a process that facilitates chromatin decondensation. This cascade was experimentally validated by our observation that pretreatment with the ROS inhibitor NAC abrogated MET generation. Furthermore, chronic macrophage activation under inflammatory conditions induces severe organelle damage. Mitochondrial impairment releases damage-associated molecular patterns (DAMPs), including mitochondrial ROS (mtROS) and mitochondrial DNA (mtDNA), which may contribute to MET formation. Concurrently, sustained organelle damage promotes hyperactivation of autophagy. Prolonged lysosomal activation increases membrane permeability, ultimately causing lysosomal rupture and MET release. Prior studies have established that the interplay between autophagy and superoxide generation is essential for chromatin de-repression during NETosis. Emerging evidence highlights the crucial role of the cytosolic DNA sensor CCDC25 in sterile inflammatory diseases, including liver fibrosis. While neutrophil extracellular traps (NETs) are established activators of the CCDC25 pathway, recent studies suggest that macrophage extracellular traps (METs) may function similarly. Specifically, it has been proposed that METs could engage the CCDC25 receptor on hepatocytes, triggering the release of pro-inflammatory factors that subsequently activate hepatic stellate cells (HSCs) and exacerbate fibrosis [12]. In line with this paradigm, our experimental data revealed a significant upregulation of CCDC25 expression in HSCs following MET stimulation. This finding implies that METs might not only act on hepatocytes but also directly or indirectly promote HSC proliferation through a potential CCDC25-dependent mechanism, thereby adding a novel dimension to the pro-fibrotic network in the liver.

Lysosomes—containing diverse hydrolytic enzymes capable of degrading proteins, nucleic acids, and other macromolecules—play critical roles in cellular autophagy and defense. Macrophages rely heavily on lysosomes for digesting foreign material [42, 43]. While studies report lysosomal activation in macrophages stimulated by various agents and cytokines (e.g., IL-1α, IL-6, TNF-α), prolonged inflammatory activation can lead to organelle damage and subsequent autophagy. Key findings from our study reveal that despite early lysosomal activation by PMA in macrophages, sustained stimulation for 12 h resulted in a decrease in active lysosomes, coinciding with increased MET generation. Furthermore, cellular fluorescence staining identified regions mutually exclusive for MET formation and active lysosomes, demonstrating a direct link between lysosomal damage and MET generation. This critical role of lysosomes was further corroborated by the reduction in PMA- and IL-25-induced METs following treatment with the lysosomal inhibitor chloroquine (CQ). Significantly, lysosomal rupture triggers substantial calcium ion (Ca^2^⁺) release. Given that chromatin decondensation during MET formation requires the Ca^2^⁺-dependent enzyme PAD4, future investigations should explore the potential synergistic role of IL-25 in activating PAD4, which may further drive MET formation.

Our study reveals that organs and tissues subjected to external stress exhibit spontaneous stress responses and functional dysregulation, a protective mechanism aimed at mitigating damage and restoring homeostasis. In the liver, hepatocytes—the predominant parenchymal cells—play a central role in orchestrating stress responses and cytokine secretion during liver fibrosis. Interleukins, particularly IL-25, are well-established mediators of intercellular communication and have been implicated in fibrotic progression [44–46]. Prior studies demonstrated that gut microbiota dysbiosis induces IL-25 production in colonic epithelial cells, which promotes hepatocellular carcinoma (HCC) development via selective activation of macrophages in the tumor microenvironment [47]. Building on this evidence, our investigation focused on IL-25 generation within hepatocytes. Through both in vivo and in vitro experiments, we confirmed that hepatocytes experiencing stress-induced dysfunction during liver fibrosis actively produce IL-25, which subsequently modulates immune cell activity to regulate inflammatory cascades. Previous studies have established the involvement of IL-25 and IL-17 family cytokines in modulating liver fibrosis, yet their precise mechanisms remain poorly defined, with limited literature comprehensively addressing this relationship. Building on this foundation, our study employed extensive in vivo mouse models of liver fibrosis to administer IL-25, robustly demonstrating its therapeutic potential in promoting fibrotic progression. While interleukins like IL-33 and IL-22 are well-documented in chronic inflammation, our work was particularly inspired by IL-25’s role in macrophage polarization—specifically its capacity in chronic inflammation [48]. In contrast to previous studies demonstrating that IL-25 promotes fibrosis by inducing TGF-β secretion through macrophage differentiation, our study reveals a distinct mechanism: IL-25 activates its receptor, triggering downstream NOX activation and subsequent ROS burst. The IL-25 receptors, IL-17RA and IL-17RB, initiate various downstream inflammatory pathways (e.g., JAK/STAT, NF-κB, MAPK) upon ligand binding, leading to diverse mechanisms of MET formation. In this work, we demonstrated through Western blot analysis that IL-25 stimulation induces phosphorylation of key NOX activation marker proteins and a concomitant ROS burst in macrophages. Furthermore, from the perspective of cellular autophagy, transmission electron microscopy directly revealed mitochondrial damage and autophagosome formation. Subsequently, damaged mitochondria can further amplify ROS production. This elevated ROS creates conditions conducive to MET formation. Ultimately, METs act within the fibrotic liver as both activators of HSCs and damaging agents to hepatocytes, thereby promoting the progression of liver fibrosis. Critically, the injured hepatocytes themselves can produce IL-25, establishing a feed-forward loop that further exacerbates fibrosis, culminating in irreversible hepatic damage. Our findings propose a potential therapeutic strategy: modulating systemic IL-25 levels to attenuate fibrosis progression. Notably, IL-25 exhibits dual roles in macrophages – while maintaining anti-inflammatory functions (e.g., IL-10 secretion), it also exerts profibrotic effects (e.g., TGF-β secretion and promotion of MET generation). In this study, we observed that the expression of IL-25 in liver tissue decreased at the advanced stage (8 weeks) compared to earlier phases in the CCl₄-induced liver fibrosis model. We propose that this phenomenon aligns with the dynamic pathophysiological changes during fibrosis progression. In the early stage of fibrosis, damaged hepatocytes serve as the primary source of IL-25, and its release is intended to initiate repair programs. However, with sustained and chronic injury, a large number of hepatocytes undergo apoptosis, senescence, or dysfunction, leading to the gradual depletion of the cellular source of IL-25. Concurrently, microenvironment remodeling occurs, including extensive deposition of extracellular matrix forming physical compartments and the polarization of immune cells such as macrophages toward a pro-fibrotic phenotype, which may collectively alter the production and functional network of IL-25. Therefore, the peak expression of IL-25 appears during the fibrosis progression stage, while it declines in the advanced stage characterized by matrix remodeling. This suggests that IL-25 primarily acts as an "initiator" or "accelerator" of the fibrotic process, rather than a core sustaining factor in the late maintenance phase. The time-point design of our model captured this dynamic change. Given that liver fibrosis progresses through distinct stages, and considering that the abundance of hepatocytes and macrophages may decline as fibrosis advances, future clinical approaches could involve monitoring the IL-25/macrophage ratio. This strategy would enable the implementation of stage-specific therapeutic interventions to achieve optimal treatment outcomes.

Certainly, in this study, there exists a limitation in distinguishing NETs from METs. Due to the structural similarities between them and the historical use of CitH3 as a broad marker for NETs formation, we cannot entirely rule out the potential influence of NETs in this research. We do not deny the significant role of NETs in the progression of liver fibrosis. However, our study specifically focuses on METs. Our immunofluorescence co-localization images clearly demonstrate the co-localization of F4/80 and CitH3. Furthermore, we employed a neutrophil depletion model for validation and provided evidence that METs can activate HSCs. Therefore, although NETs and METs may coexist and potentially contribute in parallel to the inflammatory milieu of liver fibrosis, our work provides compelling evidence that METs constitute a previously underappreciated effector pathway with a unique regulatory mechanism. Future studies employing cell-type-specific genetic ablation models (e.g., targeting macrophages versus neutrophils) or more advanced in situ imaging techniques will be invaluable for precisely dissecting their individual spatial and temporal contributions. Nevertheless, our findings firmly establish hepatocyte-derived IL-25-driven METs formation as a novel and significant component of the pro-fibrotic network.

## Conclusion

**Fig. 8:**
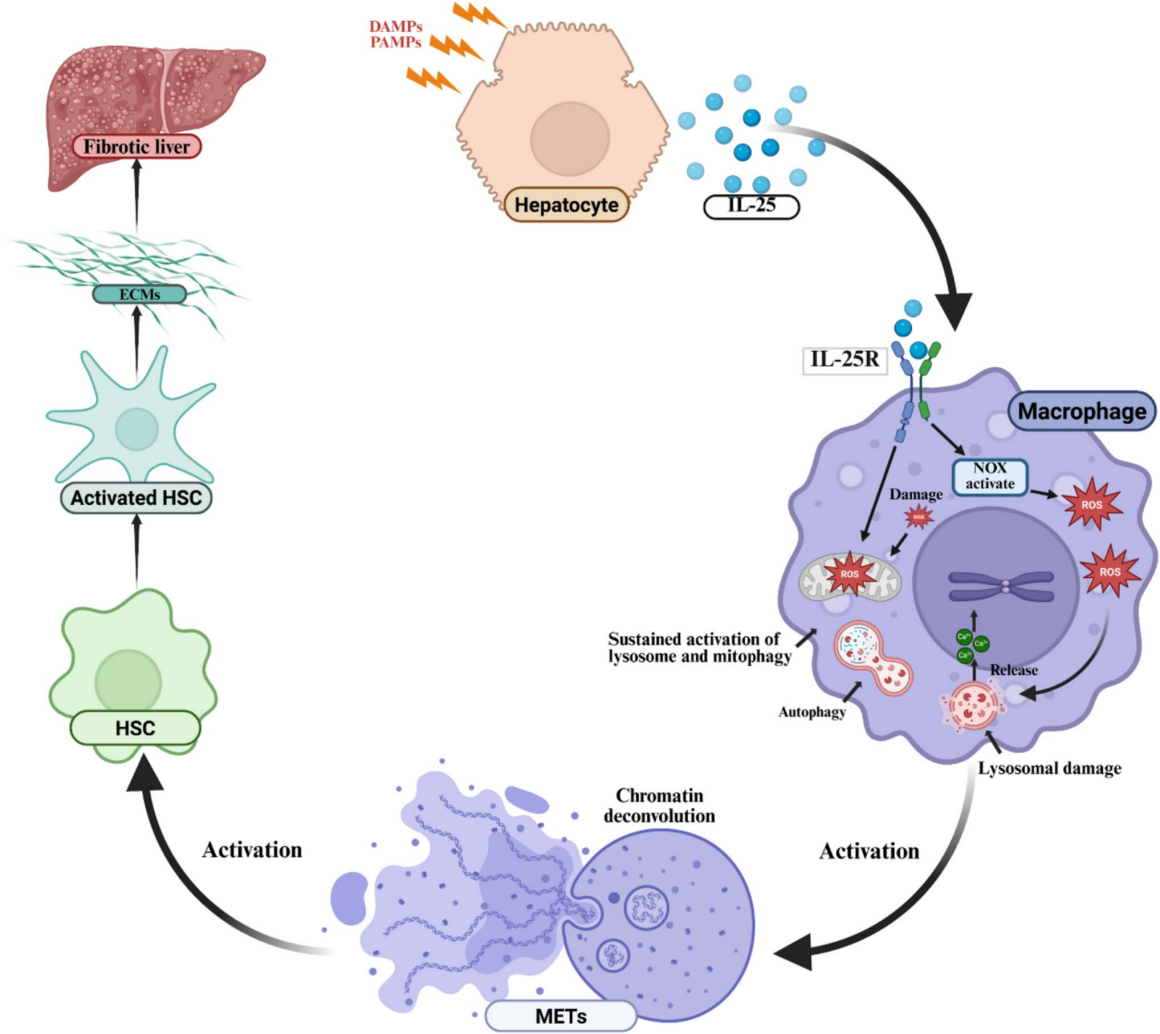
IL-25 can bind to the interleukin-25 receptor on macrophages, subsequently activating the NOX, leading to increased ROS production. Additionally, it impairs mitochondrial function, resulting in a ROS burst, triggers lysosomal dysfunction, causing calcium ion leakage from lysosomes. This, induces chromatin unraveling in macrophages, ultimately leading to macrophage rupture and the release of METs. Finally, METs activate the HSCs and causing accumulation of ECM and liver fibrosis

In summary, our study elucidates a novel mechanism for liver fibrosis regulation via the hepatocyte-macrophage-stellate cell axis: During the early stages of liver inflammation, hepatocyte-derived IL-25 acts on macrophage IL-25 receptors, inducing a ROS burst that consequently leads to MET formation. Mechanistically, this process is linked to IL-25-mediated downstream NOX activation and subsequent perturbations in mitochondrial and lysosomal stabilization (Fig. 8). Collectively, our findings confirm that controlling IL-25 production and reducing excessive MET generation during liver fibrosis represents a promising therapeutic strategy.

## Supplementary Information

Below is the link to the electronic supplementary material.

Supplementary file1 (DOCX 3051 KB)

## Data Availability

The datasets used and/or analyzed during the current study are available from the corresponding author upon reasonable request.

## Declarations

### Clinical Trial

Clinical trial number: not applicable.

## Abbreviations

α-SMA: Alpha-Smooth Muscle Actin
ALT: Alanine Aminotransferase
AST: Aspartate Aminotransferase
BMDMs: Bone Marrow-Derived Macrophages
CCK-8: Cell Counting Kit-8
CCl₄: Carbon Tetrachloride
CitH3: Citrullinated Histone H3
COL1A1: Collagen Type I Alpha 1 Chain
CQ: Chloroquine
DAMPs: Damage-Associated Molecular Patterns
DAPI: 4',6-Diamidino-2-Phenylindole
DCFH-DA: 2',7'-Dichlorodihydrofluorescein Diacetate
DNase I: Deoxyribonuclease I
ECM: Extracellular Matrix
ELISA: Enzyme-Linked Immunosorbent Assay
ETs: Extracellular Traps
HNF4α: Hepatocyte Nuclear Factor 4 Alpha
HSCs: Hepatic Stellate Cells
IL-25: Interleukin-25
LMP: Lysosomal Membrane Permeability
METs: Macrophage Extracellular Traps
NAC: N-Acetyl-L-cysteine
NOX: NADPH Oxidase
PAD4: Peptidylarginine Deiminase 4
PMA: Phorbol 12-Myristate 13-Acetate
ROS: Reactive Oxygen Species
TGF-β: Transforming Growth Factor-Beta
TNF-α: Tumor Necrosis Factor-Alpha
