# Supplementary material for: Hepatocyte-Derived IL-25 Promotes Macrophage Extracellular Trap Formation and Drives Liver Fibrosis Progression
Source: Inflammation. 2026 Apr 1;49(1):139. doi: 10.1007/s10753-026-02506-6 (PMC13171947; doi:10.1007/s10753-026-02506-6)
Supplement: Supplementary file 1 — Supplementary file1 (DOCX 3051 KB) [file 10753_2026_2506_MOESM1_ESM.docx]

**
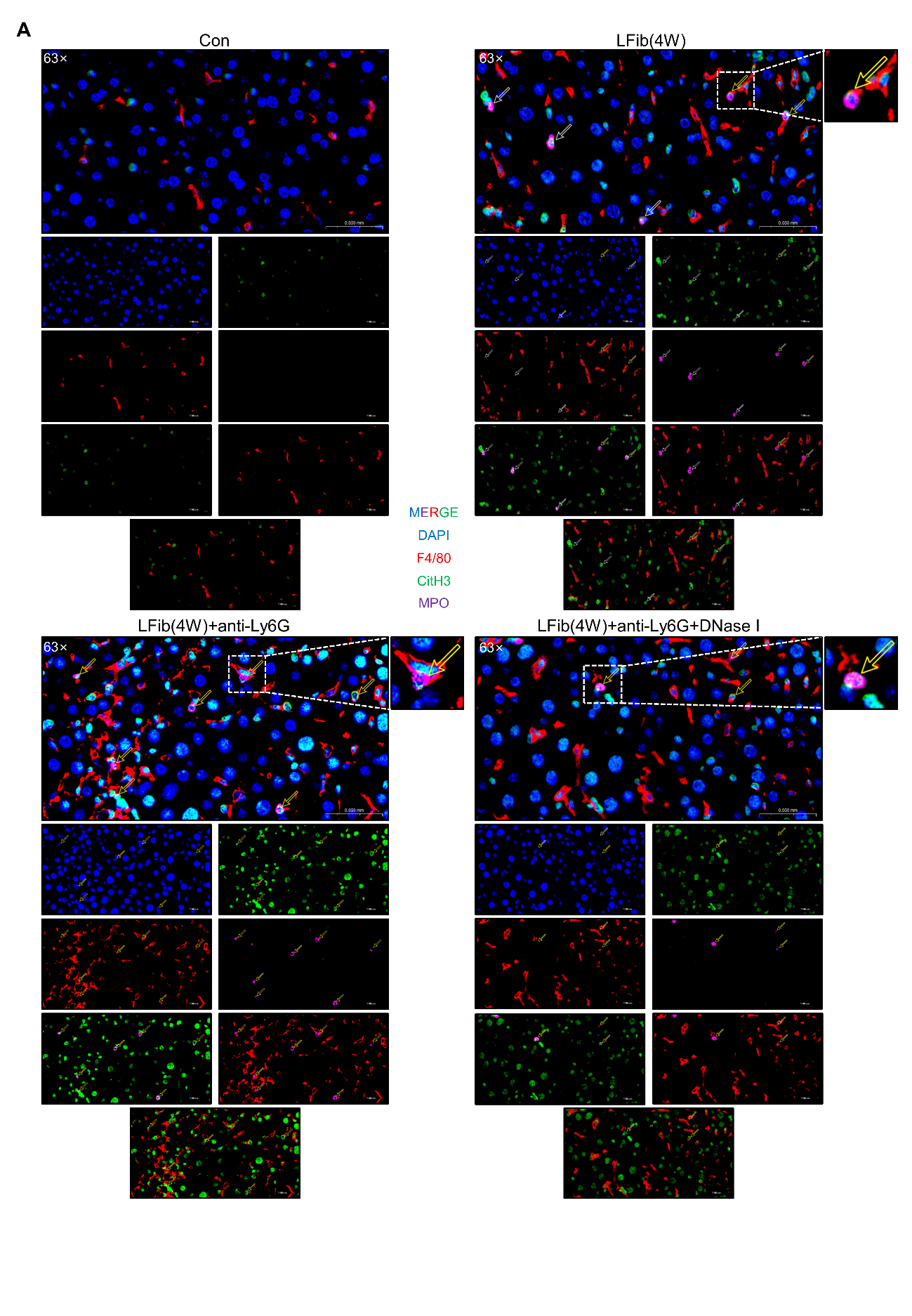
**

**Supplementary Fig. 1 (A)** Triple immunofluorescence staining of F4/80, MPO, and CitH3 in liver tissue sections from each group of mice, along with single-channel images of each fluorescence marker and selected dual-channel images. Yellow arrows indicate METs, while white arrows indicate ETs derived from other cell types. Enlarged views of representative regions are provided. Six random fields were selected per sample, with n=6 mice per group. The images shown are representative of multiple parallel experiments.

**
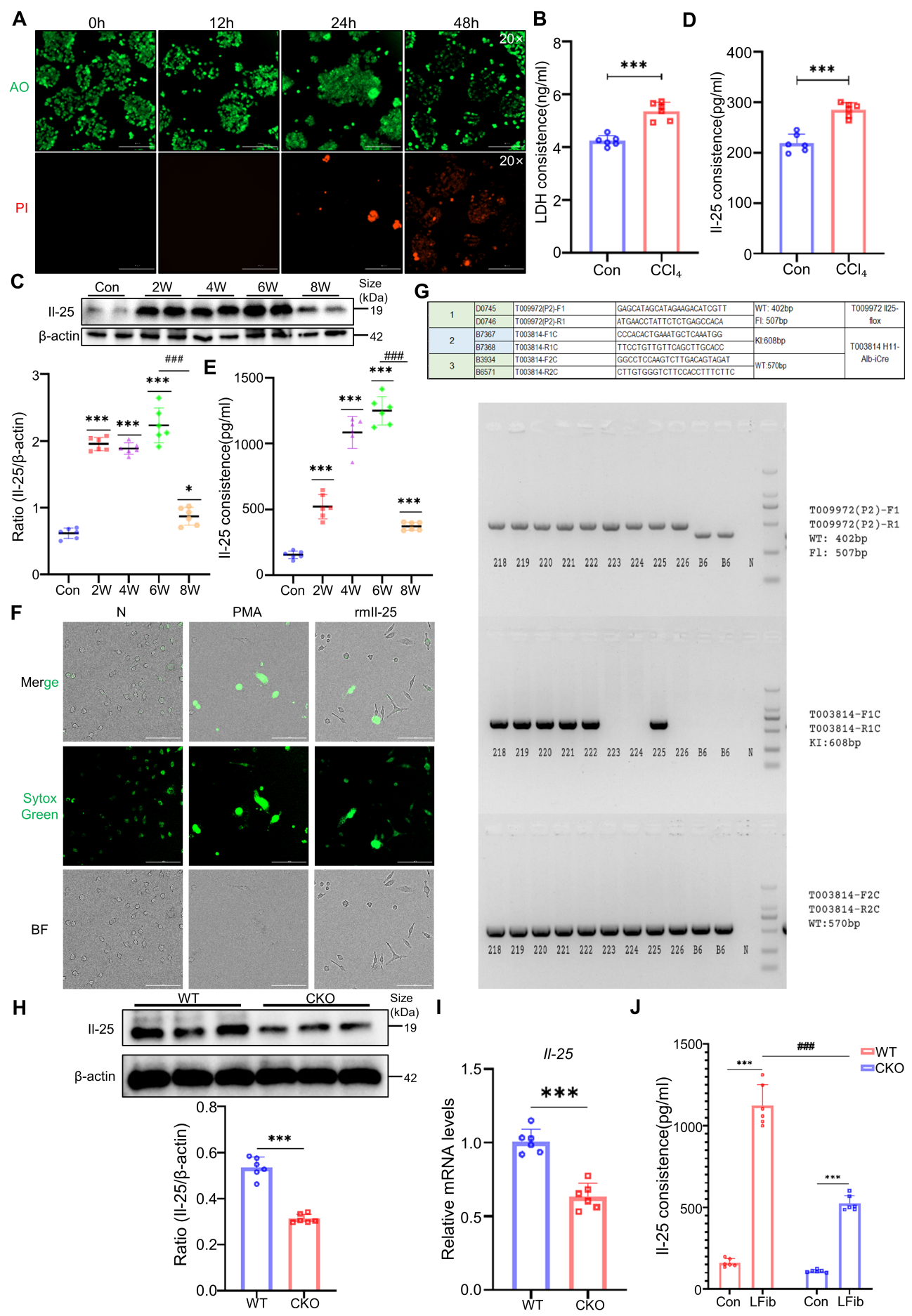
**

**Supplementary Fig. 2 (A)** AO/EB staining of AML-12 cells treated with 0.2% CCl₄ for 12h, 24h and 48h (n=3 per group). **(B)** LDH consistence in the supernatant of AML-12 cells treated with 0.2% CCl₄ for 12h, assay with LDH Kit (n=6 per group). **(C)** WB analysis of IL-25 expression in liver tissues, representative western blots from three independent experiments are shown (n=6 per group). **(D)** IL-25 consistence in the supernatant of AML-12 cells treated with 0.2% CCl₄ for 12h, assay with ELISA Kit (n=6 per group). **(E)** IL-25 consistence in the mouse peripheral blood serum, assay with ELISA Kit (n=6 per group). **(F)** Sytox Green staining images of BMDMs. (n=3 per group). **(G)** Genotyping primer sequences and representative results for IL-25CKO mice. **(H)** Protein expression of IL-25 in liver tissues of IL-25CKO mice and semi-quantification of Western blot bands, representative western blots from three independent experiments are shown (n=6 per group). **(I)** mRNA expression of *Il-25* in liver tissues of IL-25CKO mice (n=6 per group). **(J)** Serum IL-25 levels in wild-type and IL-25CKO mice in a 4-week liver fibrosis model (n=6 per group). Mean ± SEM; *p<0.05, **p<0.01, ***p<0.001, ****p<0.0001. Student’s t-test.
